# The Relationship between Habitat Loss and Fragmentation during Urbanization: An Empirical Evaluation from 16 World Cities

**DOI:** 10.1371/journal.pone.0154613

**Published:** 2016-04-28

**Authors:** Zhifeng Liu, Chunyang He, Jianguo Wu

**Affiliations:** 1 Center for Human-Environment System Sustainability (CHESS), State Key Laboratory of Earth Surface Processes and Resource Ecology, Beijing Normal University, Beijing, People's Republic of China; 2 School of Life Sciences and School of Sustainability, Arizona State University, Tempe, Arizona, United States of America; Universidade Federal de Goiás, BRAZIL

## Abstract

Urbanization results in habitat loss and habitat fragmentation concurrently, both influencing biodiversity and ecological processes. To evaluate these impacts, it is important to understand the relationships between habitat loss and habitat fragmentation per se (HLHF) during urbanization. The objectives of this study were two-fold: 1) to quantify the different forms of the HLHF relationship during urbanization using multiple landscape metrics, and 2) to test the validity of the HLHF relations reported in the literature. Our analysis was based on a long-term urbanization dataset (1800–2000) of 16 large cities from around the world. Habitat area was represented as the percentage of non-built-up area in the landscape, while habitat fragmentation was measured using several landscape metrics. Our results show that the relationship between habitat loss and habitat fragmentation during urbanization is commonly monotonic—linear, exponential, or logarithmic, indicating that the degree of habitat fragmentation per se increases with habitat loss in general. We compared our results with 14 hypothesized HLHF relationships based on simulated landscapes found in the literature, and found that four of them were consistent with those of urbanization, whereas the other ten were not. Also, we identified six new HLHF relationships when fragmentation was measured by total core area, normalized total core area, patch density, edge density and landscape shape index, respectively. In addition, our study demonstrated that the “space-for-time” approach, frequently used in ecology and geography, generated specious HLHF relationships, suggesting that this approach is largely inappropriate for analyses of urban landscapes that are highly heterogeneous in space and unusually contingent in dynamics. Our results show both generalities and idiosyncrasies of the HLHF relationship, providing new insights for assessing ecological effects of urbanization.

## Introduction

Habitat loss generally refers to the decrease in the spatial extent of natural habitat, including forest, grassland, desert, and wetlands [1, 2], whereas habitat fragmentation per se is the breaking apart of habitat after controlling for habitat loss [3]. Habitat loss and fragmentation usually occur concurrently and are interrelated, both influencing biodiversity and ecological processes and being widely concerned [2–8]. Urbanization has been accelerating around the world during the past several decades [9–12], becoming an increasingly important cause of habitat loss and fragmentation [13–19]. From 2010 to 2050, the proportion of urban population is estimated to increase from 51.6% to 67.2% around the world [20], meanwhile the built-up area will increase by 3 times [21]. During urbanization, large areas of natural habitat have been converted into impervious surfaces, causing habitat loss [17–19]. Simultaneously, the development of roads, railways, and other impervious surfaces results in habitat fragmentation per se [13, 15, 22]. To assess the impacts of urbanization on habitat, and further on biodiversity and ecosystems, understanding the relationships between habitat loss and habitat fragmentation per se during urbanization is an important and essential step [3, 23–25].

In the past few decades, several studies on the relationships between habitat loss and fragmentation have been conducted [23, 24, 26–29]. For example, Gustafson and Parker [23], as well as Pearson and Gardner [30], examined the relationships between habitat loss and fragmentation based on landscapes simulated by a percolation (or neutral) model. From empirical and theoretical studies, Fahrig [3] found that habitat loss and fragmentation were highly correlated showed significantly quadratic relationship (e.g., habitat amount and the number of patches). However, most of the relationships between habitat loss and fragmentation were derived from simulated landscapes (S1 Appendix), and few have examined real urbanizing landscapes. Since habitat loss and fragmentation during urbanization are complex and characteristic processes, caused by conversions from various habitats (e.g., forest, grassland, wetland, etc.) to built-up areas with a variety of size and shape (e.g., road, house, factory, etc.), it remains unclear whether there are some consistent relationships between habitat loss and habitat fragmentation per se during urbanization, and how they differ from the relationships based on simulated landscapes reported in the literature.

In this study, our main objectives are two-fold: 1) to quantify the different forms of the HLHF relationship during urbanization using multiple landscape metrics, and 2) to test the validity of the HLHF relations reported in the literature. To achieve these objectives, data on urbanization over a long period of 200 years (1800–2000) in the world's 16 cities were used to extract dynamics of habitat loss and fragmentation per se and quantify their relationships (see Materials and Methods for detail). In addition, the relationships between habitat loss and habitat fragmentation based on simulated landscapes reported in eight articles were summarized to compare with our results in real urbanizing landscapes (see S1 Appendix for detail).

## Materials and Methods

### Data acquisition and processing

**Fig 1:**
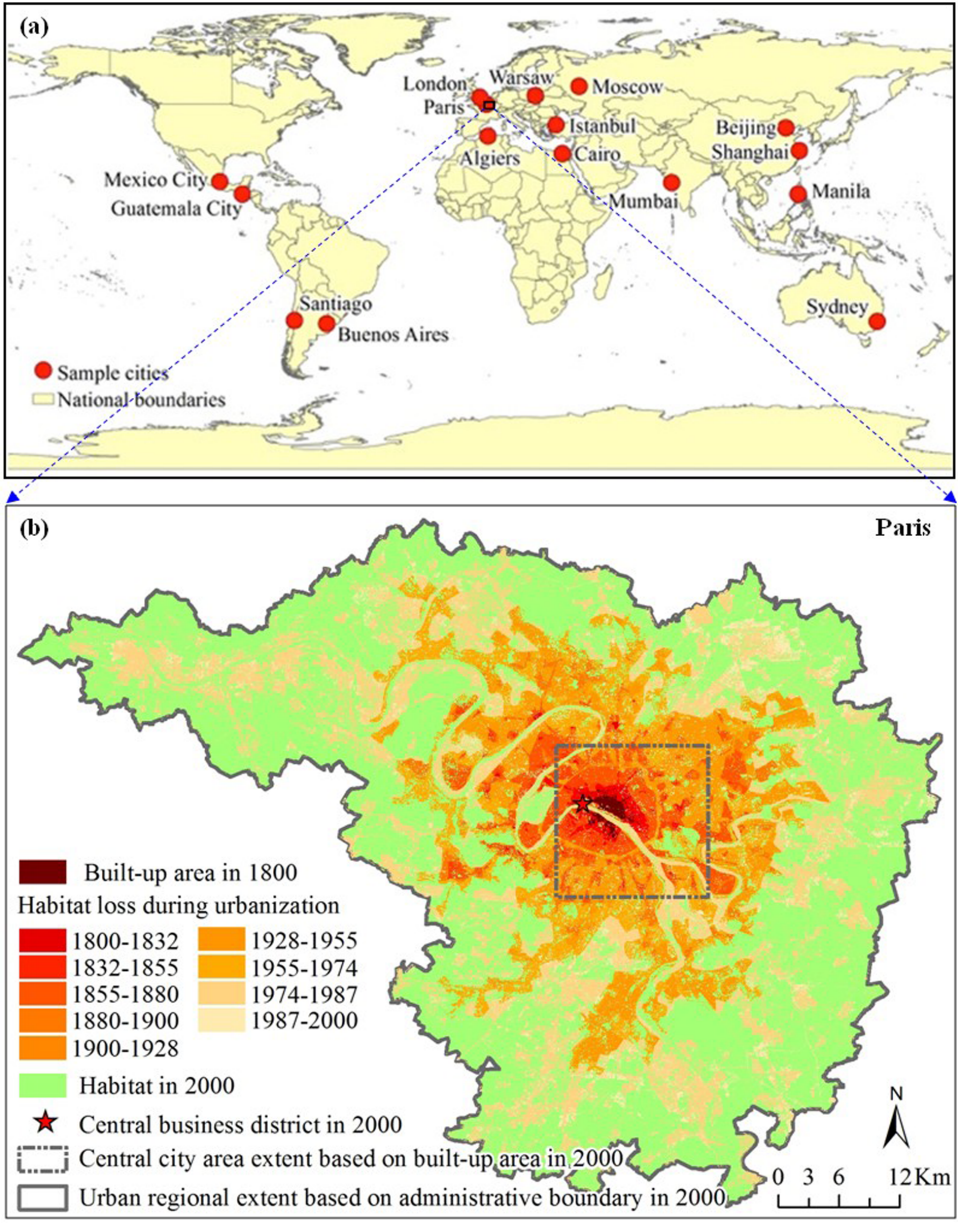
**The locations of the 16 study cities (a) and, as an example, the habitat loss during urbanization in Paris from 1800 to 2000 (b).**

The data used in our study were mainly acquired from the Dataset of Global Historical Sample of 30 Cities in the Lincoln Institute of Land Policy's Atlas of Urban Expansion (available at the institute’s website: http://www.lincolninst.edu/subcenters/atlas-urban-expansion/, accessed January 7, 2014) [31]. Specifically, we used the built-up areas, the administrative boundaries, locations of central business districts (CBD), and urban population of 16 cities around the world, i.e., Algiers, Beijing, Buenos Aires, Cairo, Guatemala City, Istanbul, London, Manila, Mexico City, Moscow, Mumbai, Paris, Santiago, Shanghai, Sydney, and Warsaw, with most complete data in the period of 1800–2000, among the 30 sample cities (Fig 1). These data were produced using both remote sensing imageries and historical maps (Angel et al. 2010) (S2 Appendix).

After the data acquisition, we implemented a time series correction for the built-up areas from 1800 to 2000 to improve their continuity and comparability, and then employed an indirect accuracy assessment based on urban population to test the consistency of the corrected data [12]. We found that the built-up area with correction was highly consistent with urban population (See Figs A and B in S3 Appendix for details), which could represent the trend of urbanization in a reliable way. The details of the time series correction and the accuracy assessment had been described in our previous study [12].

### Evaluation of the relationship between habitat loss and fragmentation

Our method for evaluating the relationships between habitat loss and habitat fragmentation per se during urbanization included three steps: 1) extracting habitats; 2) quantifying habitat fragmentation per se; 3) analyzing relationships between habitat loss and habitat fragmentation per se.

First of all, in consideration of data availability, we regarded all the non-built-up areas as habitats—similar to the habitats in the wider sense defined by IUCN—including forest, grassland, wetlands, cropland, and so forth [1]. Then, the habitats were extracted in each city from 1800 to 2000 (Fig 1).

At the second step, we chose several landscape metrics used in previous studies to quantify habitat fragmentation per se for facilitating comparison [23, 24, 26–29]. Particularly, we selected ten landscape metrics within four groups including: (1) area metrics, i.e., mean patch size (MPS), total core area (TCA), and normalized TCA (NTCA); (2) density metrics, i.e., patch density (PD) and edge density (ED); (3) shape metrics, i.e., landscape shape index (LSI) and perimeter-area fractal dimension (PAFD); and (4) connectivity metrics, i.e., mean Euclidean nearest neighbor distance (NND), normalized NND (NNND), and cohesion (Table 1) [32–33].

**Table 1:** List of landscape metrics used in the study, all of which, except normalized total core area and normalized nearest neighbor distance, were based on McGarigal et al. [32] and Wu et al. [33].

Type	Landscape metric	Abbreviation	Description
Area metrics	Mean Patch Size*	MPS	The average area of all patches in the landscape (unit: ha).
	Total Core Area*	TCA	The sum of the core areas of each patch of the corresponding patch type (unit: ha).
	Normalized TCA**	NTCA	The TCA normalized by habitat abundance.
Density metrics	Patch Density*	PD	The number of patches per square kilometer (i.e., 100 ha).
	Edge Density*	ED	The total length of all edge segments per hectare for the class or landscape of consideration (unit: m/ha).
Shape metrics	Landscape Shape Index*	LSI	A modified perimeter-area ratio of the form that measures the shape complexity of the whole landscape or a specific patch type.
	Perimeter-Area Fractal Dimension*	PAFD	An index that reflects shape complexity across a range of spatial scales (patch sizes).
Connectivity metrics	Mean Euclidean Nearest Neighbor Distance*	NND	The distance to the nearest neighboring patch of the same type, based on shortest edge-to-edge distance (unit: m).
	Normalized NND**	NNND	The NND normalized by habitat abundance.
	Cohesion*	Cohesion	An index that measures the physical connectedness of the corresponding patch type.

* The mathematical formulations can be found in McGarigal et al. [32].

** The mathematical formulations can be found in Wang and Cumming [29].

After that, we carried out two commonly used approaches—historical data based approach and space-for-time based approach [23, 28, 29, 34]—to evaluate relationships between habitat loss and habitat fragmentation per se. First, we performed the historical analysis at two extents, including the smaller extent of central city area dominated by built-up area [see details in 12] and the larger extent of urban region defined by administrative boundaries [11, 12] (Fig 1B), to explore possible effects of changing spatial scales [35, 36]. At two extents, we respectively developed regression models of different types of function for habitat proportion and each landscape metric using historical data of habitat in each city from 1800 to 2000. The regression model with the highest value of *R*^*2*^ was selected for representing the relationship between the habitat amount and the corresponding landscape metric. Based on space-for-time perspective, we built three types of grid with different spatial extents (i.e., 64 by 64 pixels or about 2 km by 2 km, 128 by 128 pixels or about 4 km by 4 km, and 256 by 256 pixels or about 8 km by 8 km) to test the scale effects found in relevant studies [27, 28, 37] (See Fig C in S3 Appendix for details). At three extents, we calculated the habitat proportion and the ten landscape metrics in each grid in each city in 2000, and then analyzed relationships between habitat proportion and landscape metrics using their values for all the grids. All of the landscape metrics were computed using the FRAGSTATS software (v4.1) [32].

In addition, we reviewed 22 relevant papers between 1992 and 2015 to summarize hypotheses on relationship between habitat loss and fragmentation (Text A in S1 Appendix). Among them, eight papers reported the relationships between habitat loss and fragmentation based on simulated landscapes [7, 23, 26–30, 38], and we summarized them into 14 forms (Figs A–J in S1 Appendix). By comparing these relationships in simulated landscapes with our results in real urbanizing landscapes, we classified the 14 forms into the following three groups: relationships found in both simulated landscapes and real urbanizing landscapes, relationships only found in real urbanizing landscapes, and relationships only found in simulated landscapes.

## Results

### General trend of habitat loss during urbanization

**Fig 2:**
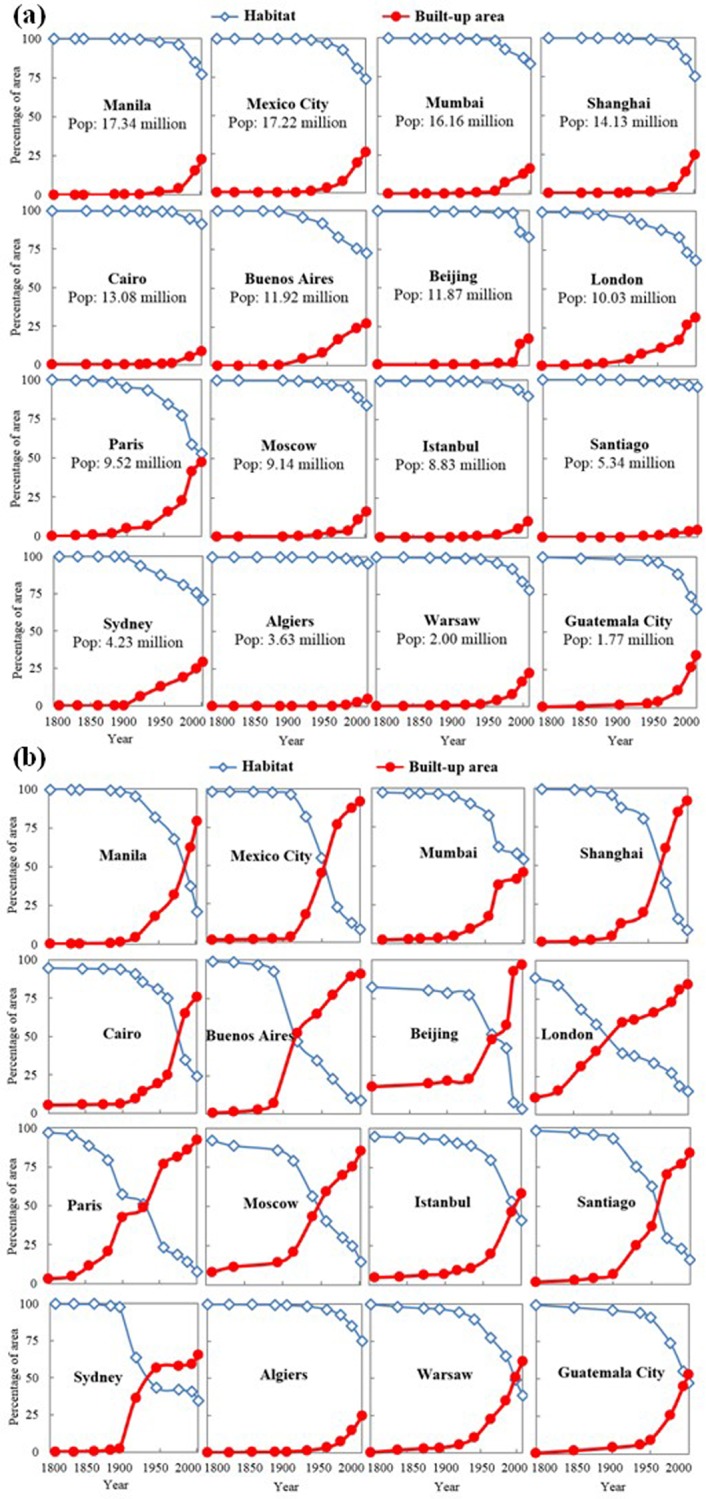
**Changes in built-up area and habitat in 16 study cities from 1800 to 2000 at two extents: (a) the urban region and (b) the central city area.** The study cities are ordered by urban population in 2000.

As urbanization unfolded, habitat area decreased slowly during the first century, and then accelerated rapidly since about 1900 for all the 16 cities, while built-up area showed an opposite trend (Fig 2). This general pattern was consistent for the two spatial extents (i.e., the urban region and the central city area). Specifically, from 1800 to 1900 only less than 5% of habitat was lost for both spatial extents, but from 1900 to 2000 about 25% of habitat was lost at the urban regional scale (Fig 2A) and more than 75% was lost at the central city scale (Fig 2B) in general. In the following section, we describe how habitat fragmentation, as measured by 10 different landscape metrics, changed with habitat loss.

### Habitat loss-fragmentation relationship based on historical urbanization data

**Fig 3:**
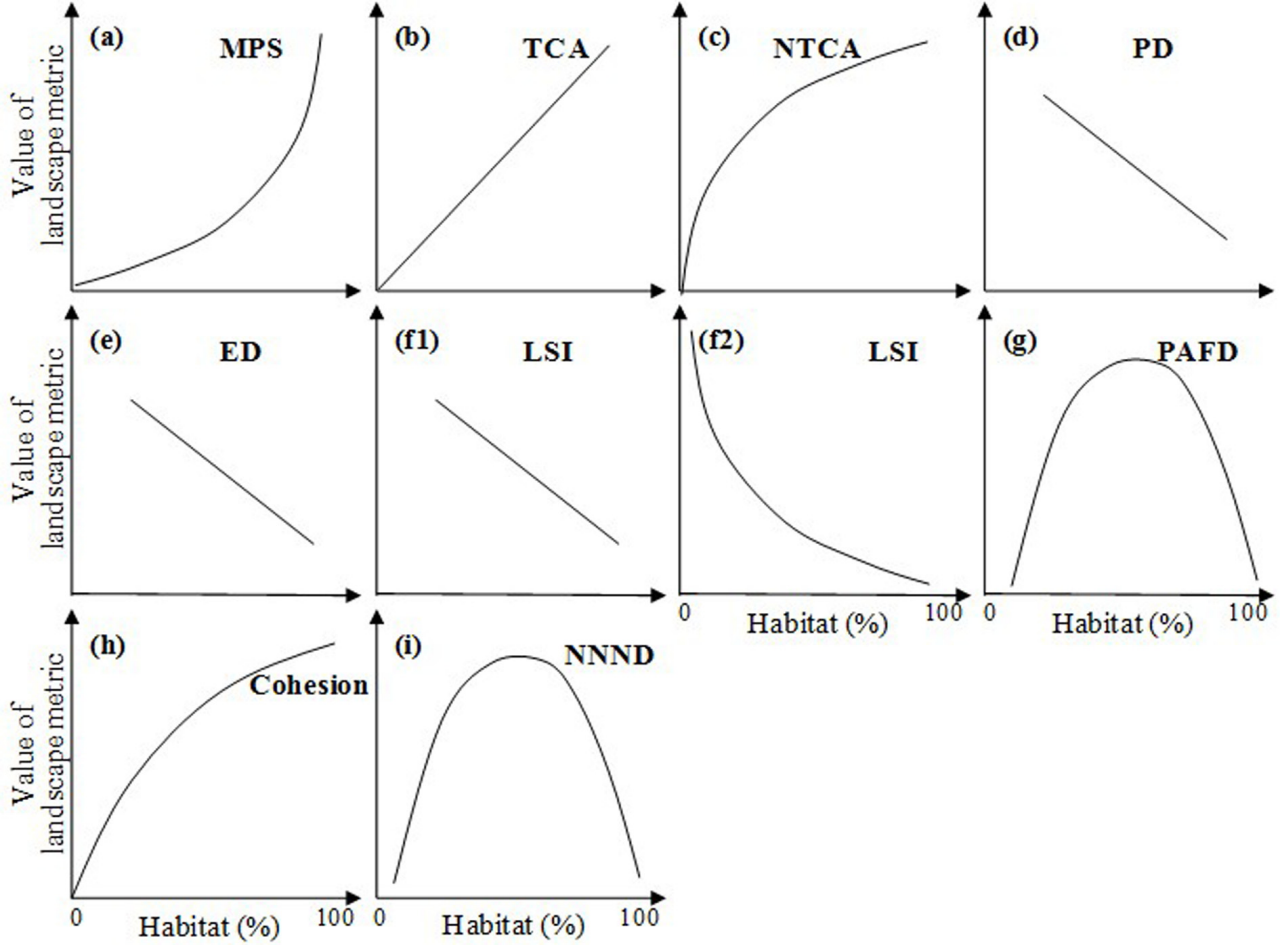
Different forms of the relationship between habitat loss and habitat fragmentation during urbanization, derived from historical landscape pattern analysis (See Figs A–J in S4 Appendix for details). *Landscape metrics include: (1) area metrics, i.e., mean patch size (MPS), total core area (TCA), and normalized TCA (NTCA); (2) density metrics, i.e., patch density (PD) and edge density (ED); (3) shape metrics, i.e., landscape shape index (LSI) and perimeter-area fractal dimension (PAFD); and (4) connectivity metrics, i.e., mean Euclidean nearest neighbor distance (NND), normalized NND (NNND), and Cohesion (See Table 1 for details).

In the period of 1800–2000, the percentage of habitat was significantly correlated with nine landscape metrics measuring habitat fragmentation per se, with *R*^*2*^ greater than 0.6 (*P*<0.01), in all the 16 world cities at the central city area extent and in most cases at the urban regional extent (Fig 3, Table 2, Figs A–J in S4 Appendix).

**Table 2:** Different forms of the relationship between habitat loss and habitat fragmentation during urbanization in Paris, as an example, derived from historical landscape pattern analysis at the urban regional extent (Number of samples = 10) (See Figs A–J in S4 Appendix for details).

Landscape metric	Type of relationship	Formula***	*R*^*2*^
MPS	Exponential	y = 0.001e^12.61x^	0.72*
TCA	Linear	y = 530,162.42x - 216,779.52	0.99**
NTCA	Logarithmic	y = 0.99ln(x) + 1.03	0.94**
PD	Linear	y = -44.95x + 44.82	0.96**
ED	Linear	y = -205.01x + 201.45	0.95**
LSI	Linear	y = -385.27x + 378.81	0.94**
PAFD	Quadratic	y = -1.13x^2^ + 1.51x + 0.87	0.67*
Cohesion	Logarithmic	y = 0.63ln(x) + 100.00	0.98**
NND	Quadratic	y = 139.94x^2^–189.69x + 120.59	0.72*
NNND	Quadratic	y = -4.22x^2^ + 5.55x - 1.27	0.98**

*P<0.01

**P<0.001

*** x represents the percentage of habitat, y represents the value of landscape metric measuring habitat fragmentation.

Area metrics—mean patch size, total core area, and normalized total core area—were monotonically correlated with the percentage of habitat (Fig 3A–3C, Table 2). The values of mean patch size decreased exponentially with continuing habitat loss over the 200 years (Fig 3A, Table 2), while the values of total core area linearly decreased (Fig 3B, Table 2) and the values of normalized total core area decreased with a logarithmic curve (Fig 3C, Table 2).

Density metrics, i.e., patch density and edge density, and the percentage of habitat represented linear relationships (Fig 3D and 3E, Table 2). With the process of habitat loss, patch and edge density both increased linearly from 1800 to 2000.

Among two shape metrics, landscape shape index continuously increased with habitat loss. Specifically, both the linear function and the exponential function were found in terms of the relationships between landscape shape index and the percentage of habitat (Fig 3F1 and 3F2, Table 2). In addition, fractal dimension generally increased at the beginning of habitat loss, then peaked and finally decreased with continuing habitat loss, revealing quadratic relationships with the percentage of habitat (Fig 3G, Table 2).

Cohesion and normalized nearest neighbor distance—two connectivity metrics—showed logarithmic and quadratic relationships with the percentage of habitat respectively (Fig 3H and 3I, Table 2). With the habitat loss from 1800 to 2000, cohesion decreased monotonically (Fig 3H, Table 2), while normalized nearest neighbor distance increased at the first and then decreased (Fig 3I, Table 2).

In addition to the general relationships, several cities and landscape metrics showed some idiosyncratic relationships. For example, when nearest neighbor distance was used to indicate habitat fragmentation per se, the significant relationships were not found in most cases (Fig I in S4 Appendix). Also, fractal dimension in Beijing and London did not reveal the quadratic functions, which were found in other cities at the central city area extent (Fig G-b in S4 Appendix).

### Habitat loss-fragmentation relationship based on space-for-time analysis

**Fig 4:**
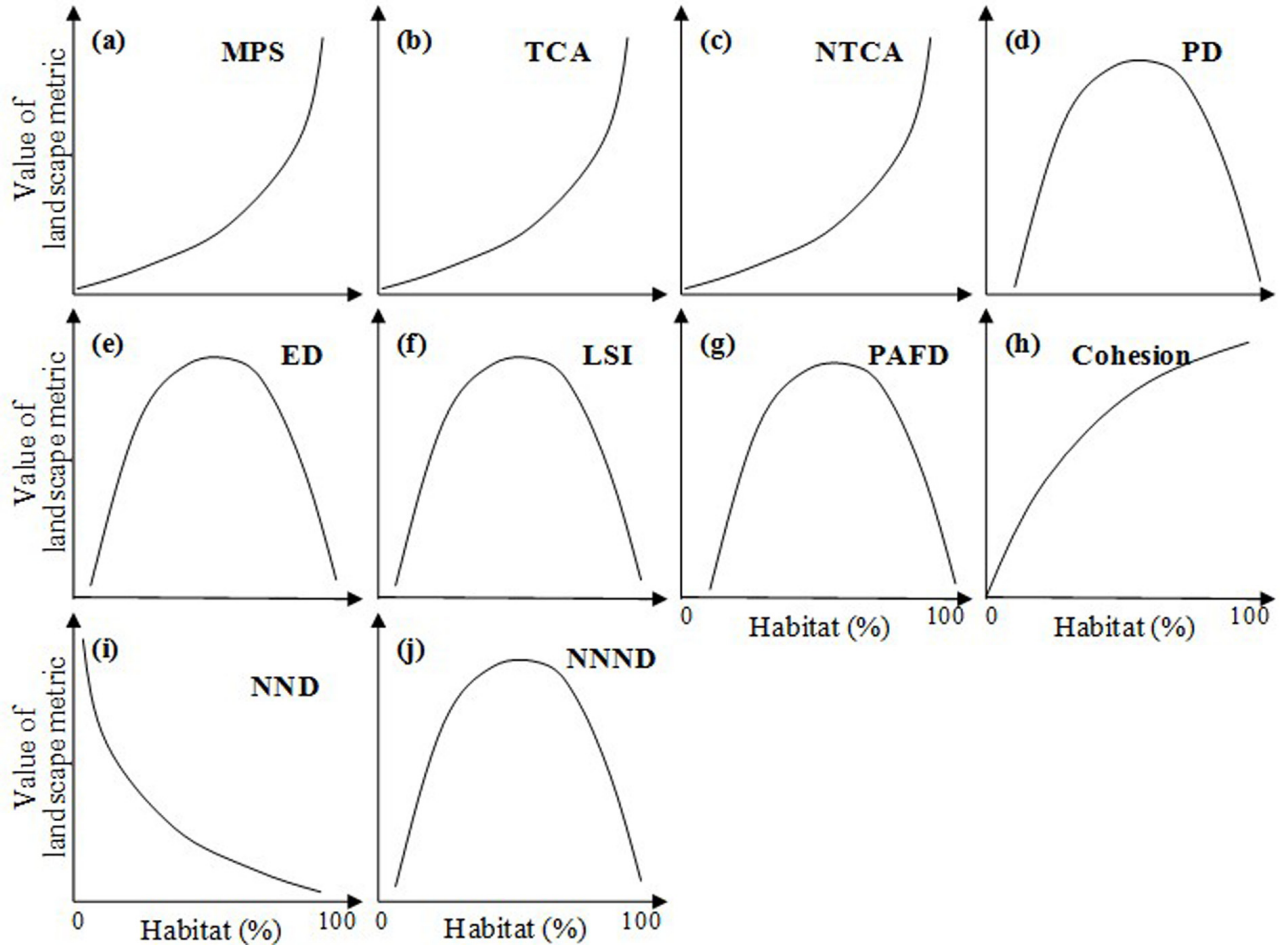
Different forms of the relationship between habitat loss and habitat fragmentation during urbanization, derived from space-for-time analysis (See Figs K–AD in S4 Appendix for details). *Landscape metrics include: (1) area metrics, i.e., mean patch size (MPS), total core area (TCA), and normalized TCA (NTCA); (2) density metrics, i.e., patch density (PD) and edge density (ED); (3) shape metrics, i.e., landscape shape index (LSI) and perimeter-area fractal dimension (PAFD); and (4) connectivity metrics, i.e., mean Euclidean nearest neighbor distance (NND), normalized NND (NNND), and Cohesion (See Table 1 for details).

Based on the space-for-time analysis in 2000, we found that all the ten metrics measuring habitat fragmentation revealed significant correlations with the percentage of habitat, and these relationships were exactly the same while different sample extent sizes (64 by 64 pixels, 128 by 128 pixels, and 256 by 256 pixels) were used in the 16 world cities (Fig 4, Table 3, Figs K–AD in S4 Appendix).

**Table 3:** Different forms of the relationship between habitat loss and habitat fragmentation during urbanization in Paris, as an example, derived from space-for-time analysis at the extent of 64 by 64 pixels (Number of samples = 818) (See Figs K–T in S4 Appendix for details).

Landscape metric	Type of relationship	Formula**	*R*^*2*^
MPS	Exponential	y = 0.17e^5.35x^	0.89*
TCA	Exponential	y = 0.63e^6.28x^	0.75*
NTCA	Exponential	y = 0.02e^3.64x^	0.55*
PD	Quadratic	y = -91.00x^2^ + 62.14x + 20.23	0.65*
ED	Quadratic	y = -442.76x^2^ + 459.54x + 18.72	0.72*
LSI	Quadratic	y = -52.87x^2^ + 35.21x + 21.32	0.63*
PAFD	Quadratic	y = -0.32x^2^ + 0.30x + 1.29	0.16*
Cohesion	Logarithmic	y = 14.12ln(x) + 103.84	0.84*
NND	Power	y = 46.75x^-0.24^	0.76*
NNND	Quadratic	y = -1.07x^2^ + 0.68x + 0.38	0.88*

* *P*<0.001

** x represents the percentage of habitat, y represents the value of landscape metric measuring habitat fragmentation.

Three area metrics, including mean patch size, total core area, and normalized total core area, all decreased exponentially with habitat loss (Fig 4A–4C, Table 3). In addition, density metrics (i.e., patch density and edge density) and shape metrics (i.e., landscape shape index and fractal dimension) increased first, then peaked and finally decreased, showing quadratic relationships with the percentage of habitat (Fig 4D–4G, Table 3). Among three connectivity metrics, cohesion and nearest neighbor distance respectively revealed positively logarithmic and negatively power relationships with the percentage of habitat (Fig 4H and 4I, Table 3), whereas normalized nearest neighbor distance and the percentage of habitat represented quadratic relationships (Fig 4J, Table 3).

### Hypothesis testing with historical data

**Fig 5:**
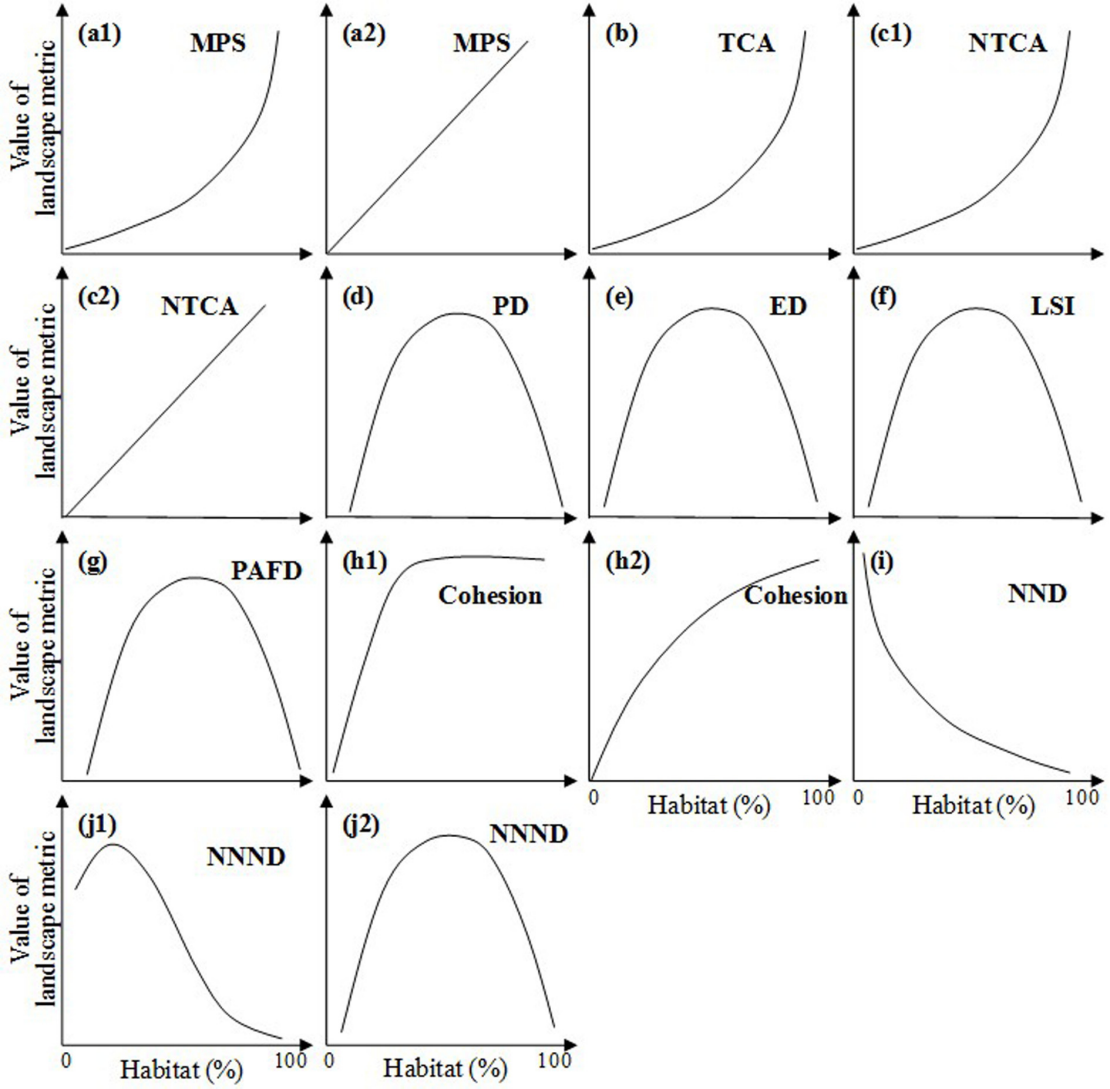
Different forms of the relationship between habitat loss and habitat fragmentation, representing different hypotheses reported in the literature (See S1 Appendix for the detailed sources). *Landscape metrics include: (1) area metrics, i.e., mean patch size (MPS), total core area (TCA), and normalized TCA (NTCA); (2) density metrics, i.e., patch density (PD) and edge density (ED); (3) shape metrics, i.e., landscape shape index (LSI) and perimeter-area fractal dimension (PAFD); and (4) connectivity metrics, i.e., mean Euclidean nearest neighbor distance (NND), normalized NND (NNND), and Cohesion (See Table 1 for details).

Four forms of the HLHF relationship were shared by simulated landscapes and real urbanizing landscapes. These consistent relationships included exponential relationships between mean patch size and the percentage of habitat (Fig 3A, Fig 5A1), logarithmic relationships between the percentage of habitat and cohesion (Figs 3H and 5H2), and quadratic relationships between the percentage of habitat and fractal dimension and normalized nearest neighbor distance (Figs 3G, 3I, 5G and 5J2).

Other ten forms of relationship were only found in simulated landscapes (Fig 5). For instance, the quadratic relationships between the percentage of habitat and density metrics (patch density and edge density) (Fig 5D and 5E), the relationships between the percentage of habitat and the area metrics of total core area and normalized total core area (Fig 5B, 5C1 and 5C2), the relationships between the percentage of habitat and the shape metric of landscape shape index (Fig 5F), and the relationships between the percentage of habitat and the connectivity metric of nearest neighbor distance (Fig 5I).

In addition, six new forms of relationship were found in urbanizing landscapes, i.e., a linear relationship between the percentage of habitat and total core area (Fig 3B), a logarithmic relationship between the percentage of habitat and normalized total core area (Fig 3C), and a negatively monotonic relationship between the percentage of habitat and density metrics of patch density and edge density and between the percentage of habitat and landscape shape index (Fig 3D–3F2).

### Hypothesis testing with space-for-time analysis

All the ten forms of habitat loss-fragmentation relationship based on space-for-time analysis are found in the literature based on simulated landscapes (Figs 4 and 5). The consistent forms included the exponential relationships between the percentage of habitat and area metrics (mean patch size, total core area, and normalized total core area), the quadratic relationships between the percentage of habitat and five metrics (i.e., density metrics of patch density and edge density, shape metrics of landscape shape index and fractal dimension, and the connectivity metric of normalized nearest neighbor distance), the logarithmic relationship between the percentage of habitat and cohesion, and the power relationship between the percentage of habitat and nearest neighbor distance (Figs 4 and 5). Moreover, other four forms of relationship, e.g., linear relationships between the percentage of habitat and two metrics (mean patch size and normalized total core area), were only demonstrated in simulated landscapes.

## Discussion

### Generalities and idiosyncrasies of the habitat loss-fragmentation relationship

Overall, the 16 study cities revealed similar relationships between habitat loss and fragmentation during urbanization. From 1800 to 2000, the continuing habitat loss in 16 cities resulted in decreases in mean patch size, total core area, normalized total core area, cohesion, and increases in patch density, edge density, and landscape shape index, suggesting increasing habitat fragmentation and shape complexity, and decreasing habitat connectivity (Fig 3).

Besides the general relationships, some idiosyncrasies existed in several cities as well. For example, Beijing and London did not reveal the quadratic function between fractal dimension and the percentage of habitat at the central city area extent (Fig G-b in S4 Appendix). It may result from that the percentage of habitat in the two cities had been still less than 90% during the study period at the central city area extent (Fig G-b in S4 Appendix), which could not reflect the sharp decrease of fractal dimension after the percentage of habitat beyond 90% in other cities (Fig G-b in S4 Appendix).

In addition, the various urbanization patterns might result in idiosyncrasies on HLHF relationships. For example, the urban land changed slightly in Beijing from 1800 to 1949, which was restricted by the city walls [39]. The particular urbanization patterns might constrain the habitat loss and fragmentation in Beijing during that period (Fig 2). The abnormal process of habitat fragmentation in Mumbai in the period of 1931–1955 might be attributable to the land policy change after the independence of India in 1947 [40]. It implied that the relevant policies on land use would be important roles in quantifying the HLHF relationships during urbanization.

### Suggestions for choosing landscape metrics in quantifying the habitat loss-fragmentation relationship

It is well known that many landscape metrics are closely related and several metrics are often endogenously correlated with habitat abundance, resulting redundancy and inaccuracy in quantifying HLHF relationships [29, 32]. To eliminate related metrics and quantify habitat fragmentation without redundancy, Frohn and Hao [41] classified 16 landscape metrics into four individual groups (i.e., class metrics, shape metrics, patch metrics and edge metrics), and found that LSI, square pixel (SqP), edge density, patch density and nearest neighbor distance were appropriate to measure habitat fragmentation. To remove the endogenous correlations between landscape metrics and habitat abundance, Wang and Cumming [29] proposed an approach to normalize landscape metrics by habitat abundance, and found that the normalization markedly reduced correlations with habitat abundance on natural landscapes.

In our research, we selected 10 landscape metrics to quantify habitat fragmentation from the literature on HLHF relationship to facilitate comparison. Specifically, three area metrics (mean patch size, total core area and normalized total core area), two density metrics (patch density and edge density), two shape metrics (landscape shape index and fractal dimension), and three connectivity metrics (nearest neighbor distance, normalized nearest neighbor distance and cohesion) were used.

We found that the choice of landscape metrics matters in quantifying the habitat loss-fragmentation relationship during urbanization. For example, total core area and the percentage of habitat were linearly correlated (Fig 3B), while nearest neighbor distance was not significantly correlated with the percentage of habitat (Fig I in S4 Appendix). After normalizing by habitat abundance [29], normalized total core area and the percentage of habitat showed logarithmic relationships (Fig 3C), and normalized nearest neighbor distance and the percentage of habitat revealed quadratic relationships (Fig 3I). In addition, fractal dimension and normalized nearest neighbor distance—two metrics calculated using values at patch level [32]—represented different relationships compared with landscape shape index and cohesion, which were directly calculated at class level (Fig 3F–3I).

To avoid redundancy and endogenous correlations, we suggested that the normalized metrics and at least one of metrics representing different aspects of habitat fragmentation should be selected in terms of the previous studies [29, 41]. In addition, two or three metrics representing habitat fragmentation in the same aspects could be used for confirming each other as well. Thus, four metrics (i.e., normalized total core area, normalized nearest neighbor distance, patch density and landscape shape index), which described habitat fragmentation in various aspects, should be selected. Mean patch size, edge density, fractal dimension and cohesion were recommended to confirm these in quantifying HLHF relationships.

### Comparing historical analysis with space-for-time analysis

In this study, the habitat loss-fragmentation relationships based on space-for-time analysis were different from relationships based on historical urbanization data in most cases (Figs 3 and 4). For example, patch density increased linearly with habitat loss from 1800 to 2000 (Fig 3D), whereas patch density and the percentage of habitat revealed quadratic relationships when space-for-time analysis was utilized (Fig 4D). The inconsistency of relationship was also found in terms of total core area, normalized total core area, edge density, landscape shape index, and nearest neighbor distance (Figs 3 and 4).

Space-for-time analysis is problematic when it is used to estimate relationships between habitat loss and fragmentation during urbanization. The space-for-time analysis, which "assumes that spatial and temporal variation are equivalent", is a commonly used approach to study long-term phenomena in ecology according to a series of different-aged samples [42], and has been used widely to evaluate habitat loss-fragmentation relationships during deforestation [27, 28, 37]. However, this approach has several problems when it is performed in landscapes across much environmental variance [42], e.g., urban landscapes with high heterogeneity and dramatic dynamics in composition and configuration [10, 33].

**Fig 6:**
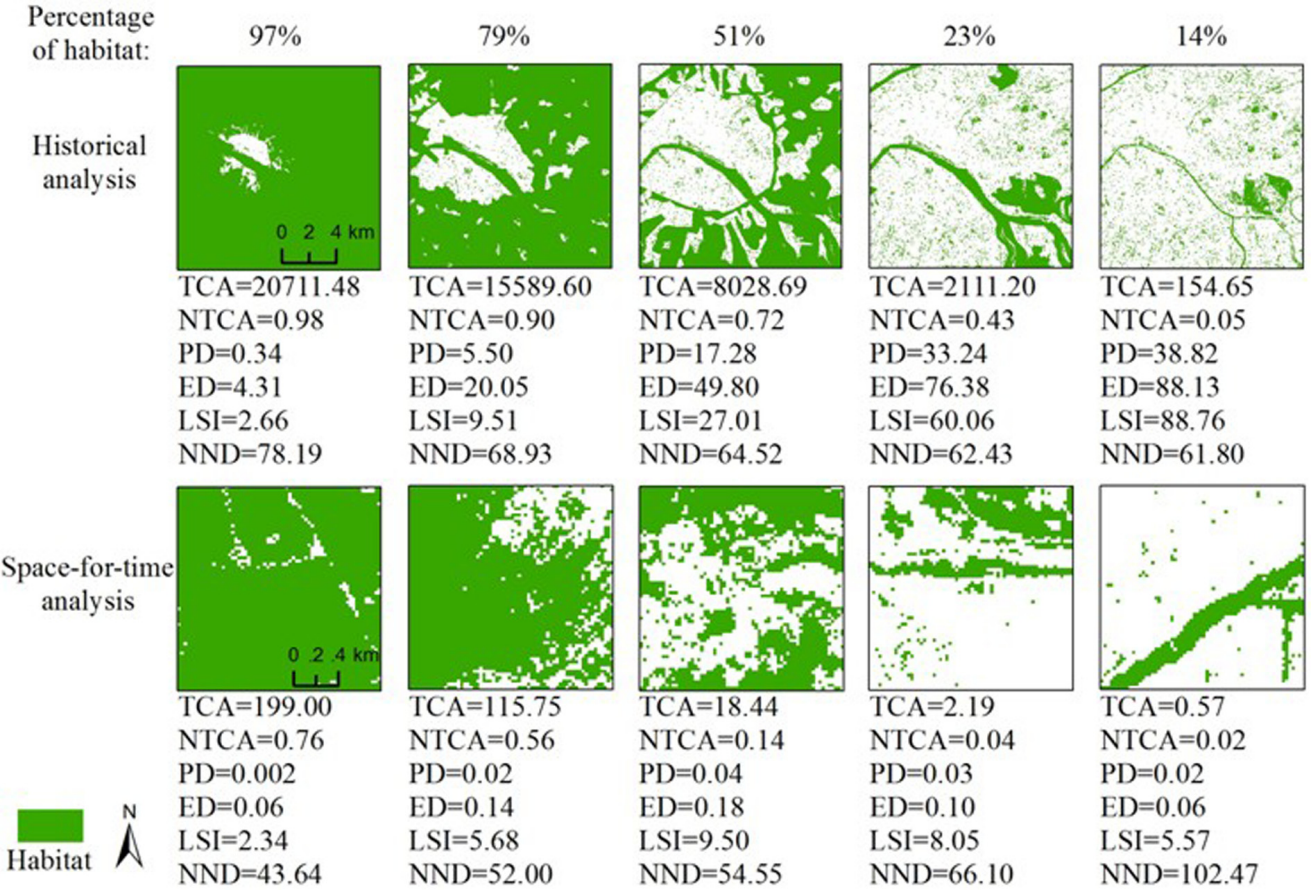
Spatial patterns of habitat and corresponding values of landscape metrics with decreasing percentages of habitat in Paris, as an example, derived from both historical landscape pattern analysis and space-for-time analysis. *The habitats in the historical analysis were derived from data at the central city area extent in Paris in 1800, 1880, 1928, 1955, and 1987. The habitats in the space-for-time analysis were derived from data in Paris in 2000 with the extent of 64 by 64 pixels.

At the central city area in Paris as an example, the patch density, edge density and landscape shape index showed linear increases with growth of 112,19 and 32 times respectively, along with the continuous decrease of the percentage of habitat from 97% to 14% (Fig 6). It represented that even the percentage of habitat was low, a large number of small patches of habitat existed in the highly urbanized area. This phenomenon was found in several cities, where many small green spaces were kept to satisfy urban residents' requirements for cultural services from urban landscapes [43]. Thus, the patch density of habitat increased even though the area of habitat decreased during urbanization. However, the space-for-time analysis based on the samples in various places cannot well capture the artificially dominated dynamics of habitat fragmentation in the process of urbanization. For instance, when the percentage of habitat decreased from 51% to 14%, the patch density, edge density and landscape shape index all declined in the areas at the extent of 64 by 64 pixels in Paris in 2000 (Fig 6). In addition, since the extent used in the space-for-time analysis is much smaller than the whole urban region, this approach would result in HLHF relationships different from the historical analysis due to the scaling effects in measuring habitat fragmentation [35, 36].

### Comparing real urbanizing landscapes with simulated landscapes

In our study, we found that four forms of the HLHF relationship were shared by real urbanizing landscapes and simulated landscapes. Among them, three forms were also consistent with that in the real landscapes with deforestation, which included exponential relationships between mean patch size and the percentage of habitat, logarithmic relationships between cohesion and the percentage of habitat, and quadratic relationships between normalized nearest neighbor distance and the percentage of habitat [23, 26, 28–30, 44]. Thus, we suggested that the three forms may represent the general HLHF relationships.

Additionally, six new forms of the HLHF relationship were found in urbanizing landscapes. Particularly, the patch density, edge density and landscape shape index showed monotonic relationships with the percentage of habitat in urbanizing landscapes, while the three metrics showed quadratic relationships with the percentage of habitat in simulated landscapes. The monotonic increases of patch density, edge density and landscape shape index along with the continuous decreases of habitat area may be attributable to the artificially dominated process of urban development (Fig 6), which cannot be well represented by landscapes simulated by percolation (or neutral) models without consideration of human activities and spatial heterogeneity [23, 30].

### Implications for mitigating urbanization effects on biodiversity

Three kinds of mechanisms have been developed for understanding effects of habitat loss and fragmentation on biodiversity[8]. First, there are those directly caused by habitat loss. Second, there are those directly caused by habitat fragmentation per se. Finally, there are those caused by indirect or interaction effects of habitat loss and fragmentation. By reviewing the literature on impacts of habitat loss and fragmentation on biodiversity, Fahrig [3] concluded that habitat loss has large effects on biodiversity, while habitat fragmentation per se has much weaker effects on biodiversity when indirect and interaction effects were ignored. However, Didham et al. [5] suggested that indirect and interaction effects of habitat loss and fragmentation can be the dominant cause of the ecological changes.

In this study, we found that habitat loss and fragmentation are significantly correlated, underlying the inherent interdependence between habitat loss and habitat fragmentation per se during urbanization. Thus, both the direct effects of habitat loss and the indirect or interaction effects of habitat loss and fragmentation should be considered for mitigating the impacts of urbanization on biodiversity. For example, the habitat loss needs to be minimized by optimizing urban form [45]. The habitat isolation and edge effects in terms of habitat loss and fragmentation can be mitigated by optimizing the density and shape complexity of habitat and increasing corridors in urban landscapes[46–48].

## Conclusions

Several conclusions can be drawn from our analysis based on long-term urbanization data. First, urbanization tends to decrease habitat amount and increase habitat fragmentation simultaneously over time. Specifically, as habitat loss continues, mean patch size, total core area, and cohesion of habitats decrease, while patch density, edge density, as well as shape complexity, all increase. This general trend holds despite the diversity of landscape metrics that are used to quantify habitat fragmentation. Second, our results indicate that, as the most intense and designed form of land use, urbanization differs in details from the simulated process of landscape modification in terms of how it influences the relationship between habitat area and fragmentation, e.g., the monotonic relationship between habitat area and shape complexity of habitat. Third, our study demonstrates that the space-for-time approach is unwarranted in evaluating the habitat loss-fragmentation relationship in urban landscapes because of their spatial heterogeneity and temporal contingency. Fourth, our results are helpful for minimizing habitat fragmentation during urbanization through explicitly optimizing the density and shape complexity of habitat in urban landscapes.

## Supporting Information

S1 Appendix The hypotheses on relationship between habitat loss and habitat fragmentation reported in the literature. (DOC)

S2 Appendix Historical map references. (DOC)

S3 Appendix Verification of data consistency and methods. (DOC)

S4 Appendix The relationships between habitat loss and habitat fragmentation during urbanization based on historical urbanization data and space-for-time analysis. (DOC)
