# Supplementary material for: The Relationship between Habitat Loss and Fragmentation during Urbanization: An Empirical Evaluation from 16 World Cities
Source: PLoS One. 2016 Apr 28;11(4):e0154613. doi: 10.1371/journal.pone.0154613 (PMC4849762; doi:10.1371/journal.pone.0154613)
Supplement: S1 Appendix — (DOC) [file pone.0154613.s001.doc]

**S1 Appendix. The hypotheses on relationship between habitat loss and habitat fragmentation reported in the literature.**

Text A. The list of papers on relationship between habitat loss and fragmentation which were reviewed (ordered by publication year).

1. Gustafson, E.J.; Parker, G.R. Relationships between landcover proportion and indices of landscape spatial pattern. Landscape Ecology 1992, 7, 101-110.

2. Andrén, H. Effects of habitat fragmentation on birds and mammals in landscapes with different proportions of suitable habitat: A review. Oikos 1994, 71, 355.

3. Robinson, S.K.; Thompson, F.R., 3rd; Donovan, T.M.; Whitehead, D.R.; Faaborg, J. Regional forest fragmentation and the nesting success of migratory birds. Science 1995, 267, 1987-1990.

4. Schumaker, N.H. Using landscape indices to predict habitat connectivity. Ecology 1996, 77, 1210-1225.

5. Fahrig, L. Relative effects of habitat loss and fragmentation on population extinction. J. Wildl. Manage. 1997, 61, 603-610.

6. Pearson, S.M.; Gardner, R.H. Neutral models: Useful tools for understanding landscape patterns. In Wildlife and landscape ecology, Springer: 1997; pp 215-230.

7. Fahrig, L. When does fragmentation of breeding habitat affect population survival? Ecol. Model. 1998, 105, 273-292.

8. Gustafson, E.J. Quantifying landscape spatial pattern: What is the state of the art? Ecosystems 1998, 1, 143-156.

9. Hargis, C.D.; Bissonette, J.A.; David, J.L. The behavior of landscape metrics commonly used in the study of habitat fragmentation. Landscape Ecology 1998, 13, 167-186.

10. Drolet, B.; Desrochers, A.; Fortin, M.J. Effects of landscape structure on nesting songbird distribution in a harvested boreal forest. Condor 1999, 101, 699-704.

11. Trzcinski, M.K.; Fahrig, L.; Merriam, G. Independent effects of forest cover and fragmentation on the distribution of forest breeding birds. Ecol. Appl. 1999, 9, 586-593.

12. Villard, M.A.; Trzcinski, M.K.; Merriam, G. Fragmentation effects on forest birds: Relative influence of woodland cover and configuration on landscape occupancy. Conserv. Biol. 1999, 13, 774-783.

13. Wickham, J.D.; Jones, K.B.; Riitters, K.H.; Wade, T.G.; O'Neill, R.V. Transitions in forest fragmentation: Implications for restoration opportunities at regional scales. Landscape Ecology 1999, 14, 137-145.

14. Belisle, M.; Desrochers, A.; Fortin, M.J. Influence of forest cover on the movements of forest birds: A homing experiment. Ecology 2001, 82, 1893-1904.

15. Boulinier, T.; Nichols, J.D.; Hines, J.E.; Sauer, J.R.; Flather, C.H.; Pollock, K.H. Forest fragmentation and bird community dynamics: Inference at regional scales. Ecology 2001, 82, 1159-1169.

16. Fahrig, L. Effects of habitat fragmentation on biodiversity. Annu. Rev. Ecol. Evol. Syst. 2003, 34, 487-515.

17. Fortin, M.J.; Boots, B.; Csillag, F.; Remmel, T.K. On the role of spatial stochastic models in understanding landscape indices in ecology. Oikos 2003, 102, 203-212.

18. Neel, M.C.; McGarigal, K.; Cushman, S.A. Behavior of class-level landscape metrics across gradients of class aggregation and area. Landscape Ecology 2004, 19, 435-455.

19. Wiegand, T.; Revilla, E.; Moloney, K.A. Effects of habitat loss and fragmentation on population dynamics. Conserv. Biol. 2005, 19, 108-121.

20. Smith, A.C.; Koper, N.; Francis, C.M.; Fahrig, L. Confronting collinearity: Comparing methods for disentangling the effects of habitat loss and fragmentation. Landscape Ecology 2009, 24, 1271-1285.

21. Wang, X.; Cumming, S.G. Measuring landscape configuration with normalized metrics. Landscape Ecology 2011, 26, 723-736.

22. Pe'er, G.; Zurita, G.A.; Schober, L.; Bellocq, M.I.; Strer, M.; Muller, M.; Putz, S. Simple process-based simulators for generating spatial patterns of habitat loss and fragmentation: A review and introduction to the g-raffe model. PLoS One 2013, 8, e64968.

Habitat Amount (%)

0

100

Patch Density

Figure A. The relationship between habitat amount and patch density of habitat from Gustafson and Parker (1992), Andrén (1994), Pearson and Gardner (1997), and Pe'er et al. (2013).

Habitat Amount (%)

0

100

Edge Density

Figure B. The relationship between habitat amount and edge density of habitat from Pearson and Gardner (1997) and Hargis et al. (1998).

Habitat Amount (%)

0

100

Landscape Shape Index

Figure C. The relationship between habitat amount and landscape shape index of habitat from Pe'er et al. (2013).

Habitat Amount (%)

0

100

Mean Patch Size

Habitat Amount (%)

0

100

Mean Patch Size

**(a)**

**(b)**

Figure D. The relationship between habitat amount and mean patch size of habitat in the literature (a: Gustafson and Parker (1992), Pearson and Gardner (1997); b: Pe'er et al. (2013)).

Habitat Amount (%)

0

100

Fractal Dimension

Figure E. The relationship between habitat amount and perimeter-area fractal dimension of habitat from Gustafson and Parker (1992) and Hargis et al. (1998).

Habitat Amount (%)

0

100

Total Core Area

Figure F. The relationship between habitat amount and total core area of habitat from Neel et al. (2004) and Wang and Cumming (2011).

Habitat Amount (%)

0

100

Normalized Core Area

Habitat Amount (%)

0

100

Normalized Core Area

**(a)**

**(b)**

Figure G. The relationship between habitat amount and normalized total core area of habitat from Wang and Cumming (2011).

Habitat Amount (%)

0

100

Nearest Neighbor Distance

Figure H. The relationship between habitat amount and nearest neighbor distance of habitat from Gustafson and Parker (1992), Andrén (1994), and Wang and Cumming (2011).

Habitat Amount (%)

0

100

Normalized Nearest Neighbor Distance

Habitat Amount (%)

0

100

Normalized Nearest Neighbor Distance

**(a)**

**(b)**

Figure I. The relationship between habitat amount and normalized nearest neighbor distance of habitat from Wang and Cumming (2011).

Habitat Amount (%)

0

100

Cohesion

Habitat Amount (%)

0

100

Cohesion

**(a)**

**(b)**

Figure J. The relationship between habitat amount and Cohesion of habitat in the literature (a: Gustafson (1998), Neel et al. (2004); b: Pe'er et al. (2013)).

**References**

Andrén H (1994) Effects of Habitat Fragmentation on Birds and Mammals in Landscapes with Different Proportions of Suitable Habitat: A Review. Oikos 71(3):355

Gustafson EJ (1998) Quantifying landscape spatial pattern: What is the state of the art? Ecosystems 1(2):143-156

Gustafson EJ, Parker GR (1992) Relationships between landcover proportion and indices of landscape spatial pattern. Landscape Ecology 7(2):101-110

Hargis CD, Bissonette JA, David JL (1998) The behavior of landscape metrics commonly used in the study of habitat fragmentation. Landscape Ecology 13(3):167-186

Neel MC, McGarigal K, Cushman SA (2004) Behavior of class-level landscape metrics across gradients of class aggregation and area. Landscape Ecology 19(4):435-455

Pe'er G, Zurita GA, Schober L et al (2013) Simple Process-Based Simulators for Generating Spatial Patterns of Habitat Loss and Fragmentation: A Review and Introduction to the G-RaFFe Model. PLoS One 8(5):e64968

Pearson SM, Gardner RH (1997) Neutral models: useful tools for understanding landscape patterns. Wildlife and Landscape Ecology. Springer, pp. 215-230

Wang X, Cumming SG (2011) Measuring landscape configuration with normalized metrics. Landscape Ecology 26(5):723-736
