# Supplementary material for: The Relationship between Habitat Loss and Fragmentation during Urbanization: An Empirical Evaluation from 16 World Cities
Source: PLoS One. 2016 Apr 28;11(4):e0154613. doi: 10.1371/journal.pone.0154613 (PMC4849762; doi:10.1371/journal.pone.0154613)
Supplement: S2 Appendix — (DOC) [file pone.0154613.s002.doc]

**S2 Appendix. Historical map references^*^**

^*^Selected from map references listed in Lincoln Institute's website <http://www.lincolninst.edu/subcenters/atlas-urban-expansion/resources.aspx>

Ordered by city name and map date

**Algiers**

1800

Interpolated from 1764 map and 1828 map. Since there was no difference in land cover between 1764 and 1828, it is safe to assume that the extent of built up area was within the city walls.

1828

Rottiers and Witdoeck, 1828. Carte de la Baie d’Alger, ses forts et ses environs [map], Historic Cities : Maps and Documents. 4 Sept. 2008. The Hebrew University of Jerusalem Department of Geography and The Jewish National and University Library. http://historic-cities.huji.ac.il/

1858

Johnson, Hugh J., 1858. Algeria/(LAlgerie) XLVI [map], Afriterra: The Cartographic Free Library. 4 Sept. 2008. Afriterra Foundation. www.afriterra.org

1888

(Author Unknown), 1888. Alger, Algerie Tunisie Plans de 1888 [map]. Profburp homepage. 30 August 2008. http://www.profburp.com/plan/

1903

Service Geographique de l’Armée (France), 1903. Environs d’Alger [map]. Profburp homepage. 30 August 2008. http://www.profburp.com/plans1903/

1929

Touring Club Italiano. 1977. Atlante Internazionale del Touring Club Italiano. Milano: Touring Club Italiano.

1955

U.S. Army Map Service, 1955. Alger and Vicinity [map]. Perry-Castañeda Library Map Collection. 4 September 2008. University of Texas Libraries. http://www.lib.utexas.edu/maps/

1972

Sgroï-Dufresne, Maria, 1986. Alger 1830-1984: Stratégies et Enjeux Urbains. Paris: Editions Recherche sur les Civilisations, pp. 12, 21, & insets.

**Beijing**

1800

Chongnian, Yan. 1987. Beijing – The Treasures of an Ancient Capital. Beijing: Morning Glory Press, p. 209.

1875

Petermann, A. 1876. Originalkarte der Ebene von Peking und des Gebircslandes im Westen und Norden der Capitale [map]. Perry-Castañeda Library Map Collection. 3 April 2009. University of Texas Libraries. http://www.lib.utexas.edu/maps/

1900

War Department, Adjutant General’s Office, Military Information Division. Map of the Pei-Ho from above Peking to its mouth. Plan of the city of Peking. [map]. Various scales. Washington D.C.: War Department, Military Information Division, 1900.

1929

Author unknown – Czech origin. 1930. Plán Pekingu [map]. Wikimedia Commons. 4 September 2008. http://commons.wikimedia.org/

1959

Westermann. 1992. Diercke Weltatlas. Braunschweig: Westermann Schulbuchverlag GmbH.

1978

Westermann. 1992. Diercke Weltatlas. Braunschweig: Westermann Schulbuchverlag GmbH.

**Buenos Aires**

1809 – 1887

Novick, Alicia; Collado Federico, and Favelukes Graciela, 2004. Urbanización de Buenos Aires [maps]. Atlas Ambiental de Buenos Aires. 30 August 2008. Gobierno de Buenos Aires, FADU, CONCEIT, AGENCIA. http://www.atlasdebuenosaires.gov.ar/

1918 – 1964

Gutman, Margarita and Hardoy, Jorge Enrique. 2007. Buenos Aires 1536 – 2006: Historia urbana del Área Metropolitana. Buenos Aires: Ediciones Infinito, pp. 352-360.

**Cairo**

1800, 1917, 1927, 1947, 1960

El Kadi, Galia. 1987. L’Urbanisation Spontanee au Caire, Fascicule de Recherches № 18, Tours: URBAMA, Institut de Géographie and Paris : O.R.S.T.O.M pp. 28 – 64.

1846

Szultz, Lt. Col. 1846. Plan général de la Ville du Kaire et des Environs [map]. Islamic Cities Historic Map Collection. 8 September 2008. University of California Berkeley, Center for Middle Eastern Studies. http://cmes.berkeley.edu

1874

Grand, P. 1874. Plan general de la Ville du Caire [map]. Islamic Cities Historic Map Collection. 8 September 2008. University of California Berkeley, Center for Middle Eastern Studies. http://cmes.berkeley.edu

1897

Rand McNally and Company, 1897. Map of the environs of Cairo [map]. David Rumsey Map Collection. 8 September 2008. http://www.davidrumsey.com

**Guatemala City**

1800, 1850, 1900, 1950 1976

Gellert, Gisela and Pinto Soria, J. C. 1990. Ciudad de Guatemala: Dos estudios sobre su evolucion urbana (1524 – 1950). Guatemala: Centro de Estudios Urbanos y Regionales: Universidad de San Carlos de Guatemala.

1936

Pola de Torroella, Federico. Plano de la Ciudad de Guatemala [map]. 1:10,000. Guatemala City: Bureau Internaciónal Tecnico de la Publicidad, 1936.

**Istanbul**

1807

Artaria et Comp. 1800. Plan De Constantinople et Du Bosphore: pour server de renseignement a la Carte des Limites des trios Empires [map]. 4 September 2008. The Hebrew University of Jerusalem Department of Geography and The Jewish National and University Library. http://historic-cities.huji.ac.il/

1840, 1872

Celikp, Zeynep. 1986. The Remaking of Istanbul: Portrait of an Ottoman City in the Nineteenth Century. Berkeley: University of California Press, p 5.

1899

Gerlach, Hans-Henning. 1997. Gesammelte Werke, Karl-May-Atlas. Bamberg: Karl

May Verlag, p. 100.

1916, 1934, 1960

Origin unknown – reference missing

**London**

1800, 1845, 1860, 1880, 1914

Clout, Hugh D. 1991. The Times London History Atlas. New York: Harper Collins, pp. 88, 156.

1830

Greenwood, Christopher and Greenwood, John. 1830. Map of London second edition [map]. Accessed 5 Sept. 2008. http://www.motco.com/Map/81003/

1929

Touring Club Italiano. 1977. Atlante Internazionale del Touring Club Italiano. Milano: Touring Club Italiano.

1955

Bartholomew, John. 1955. The Times Atlas of the World: Mid-Century Edition with an Index-Gazetteer. Boston: Houghton Mifflin Company.

**Manila**

1802

de Larse, Bernardo. 1802. Manila and its Outskirts [map] in “Manila 1571 – 1898: The West in the East, Center for Historic Studies of Public Works and Town Planning, Madrid, Spain.” Personal Journey’s of Bob Gardner 14 December 2008. http://www.aenet.org/manila-expo/discover.htm

1842

de la Yglesia, Antonio. 1842. The City of Manila and the villages beyond the walls in 1842 [map]. in “Manila 1571 – 1898: The West in the East, Center for Historic Studies of Public Works and Town Planning, Madrid, Spain.” Personal Journey’s of Bob Gardner 14 December 2008. http://www.aenet.org/manilaexpo/discover.htm

1884

Author unknown. 1884. Manila and its Districts [map] in “Manila 1571 – 1898: The West in the East, Center for Historic Studies of Public Works and Town Planning, Madrid, Spain.” Personal Journey’s of Bob Gardner 14 December 2008. http://www.aenet.org/manila-expo/discover.htm

1898

de Gamoenda, Francisco J. 1898. Plano de Manila y sus Arrables [map] Perry-Castañeda Library Map Collection. 4 September 2008. University of Texas Libraries. http://www.lib.utexas.edu/maps/

1918

Bach, John. City of Manila, Philippine Islands [map]. 1:11,000. Manila: Bureau of Commerce and Industry, 1918.

1945, 1971

Dick, Howard and Rimmer, Peter J. 2003. “Chapter 8: Archipelagic Cities: Manila and Jakarta” in Dick, Howard and Rimmer, Peter J. Cities Transport and Communications: The Integration of Southeast Asia Since 1850. New York: Palgrave MacMillan, pp. 257 – 288.

**Mexico City**

1807 – 1886

Carrera Stampa, Manuel. 1949 “Planos de la Ciudad de Mexico Desde 1521 Hasta Nuestros Dias,” in Boletín de la Sociedad Mexicana de Geografía y Estadística. Tomo 67, Num. 2-3, Mexico City: Sociedad Mexicana de Geografía y Estadística, pp. 229-467.

1910 – 1970

Espinosa-Lopez, Enrique. 2003. Ciudad de México: Compendio Cronológico de su Desarollo Urbano, 1521 – 2000. México: Instituto Politécnico Nacional.

**Moscow**

1808

Author unknown. 1808. Plan von Moskwa [map] Historic Cities : Maps and Documents. 5 Sept. 2008. The Hebrew University of Jerusalem Department of Geography and The Jewish National and University Library. http://historiccities.huji.ac.il/

1836

Society for the Diffusion of Useful Knowledge. 1836. Moscow [map] Historic Cities : Maps and Documents. 2 Dec. 2008. The Hebrew University of Jerusalem Department of Geography and The Jewish National and University Library. http://historic-cities.huji.ac.il/

1893

Murray, John. 1893. Map of Moscow [map] in: “Handbook for Travellers in Russia, Poland and Finland, Paris, 1893.” 2 Dec. 2008. Perry-Castañeda Library Map Collection. University of Texas Libraries. http://www.lib.utexas.edu/maps/

1914

Author unknown. 1914. Moscow I [map] 5 Sept. 2008. http://www.discusmedia.com/catalog.php?id=24903&profile=map

1939

Author unknown. 1939. Карта Москвы 1939 года? [map] 2 Dec. 2008 http://dolgopa.org/index.php?ids=80

1957

Central Institute for Scientific Research and Design in City-Planning of the State Civil Engineering Agency and Alekseevich, Viacheslav. 1958. Moscou; amenagement et implantation de la ville. Moscow: State Publishing House for City Planning, Architecture and Construction Materials.

1978

U.S. Defense Mapping Agency Topographic Center, 1978. Portion of Moskva sheet. Topographic Map NN 37, Series 1301 [map]. Perry-Castañeda Library Map Collection. 4 September 2008. University of Texas Libraries. http://www.lib.utexas.edu/maps/

**Mumbai**

1814, 1849, 1865

Rohatgi, Pauline, et al. 1997. Bombay to Mumbai: Changing Perspectives. Mumbai: Marg Publications.

1888

Meyer, Herman J. 1892. Meyers grosse konversations-lexicon 4th Edition, Volume 3. Wien und Leipzig: Bibliographisches Institut, p. 177.

1909

Bartholomew, J. G. 1909. City of Bombay [map]. In: “Imperial gazetteer of India,” New edition, published under the authority of His Majesty's Secretary of State for India in Council. Oxford: Clarendon Press, 1907-1909.” University of Chicago Library Map Collection: Digital South Asia Library. 19 September 2008. University of Chicago. http://dsal.uchicago.edu/reference/

1931

Bartholomew, J. G. 1931. Bombay Environs [map]. In: “Imperial gazetteer of India,” 1931 edition. Oxford: Clarendon Press, 1931. University of Chicago Library Map Collection: Digital South Asia Library. 19 September 2008. University of Chicago. http://dsal.uchicago.edu/reference/

1955

US Army Map Service. 1955. Bombay and Vicinity [map]. Perry-Castañeda Library Map Collection. 4 September 2008. University of Texas Libraries. http://www.lib.utexas.edu/maps/

1964

Come from a Bombay government planning document.

**Paris**

1800

Stockdale, J. 1800. A Plan of the City of Paris [map]. Historic Cities : Maps and Documents. 4 Sept. 2008. The Hebrew University of Jerusalem Department of Geography and The Jewish National and University Library. http://historiccities.huji.ac.il/

1832

Society for the Diffusion of Useful Knowledge. 1832. Paris Environs [map]. David Rumsey Map Collection. 5 September 2008. http://www.davidrumsey.com

1855

Geographisches Institut. 1855. Frankreich, entworfen und gezeichnet von C.F. Weiland. (with) Paris mit den Fortificationen. (with) Insel Corsica [map]. David Rumsey Map Collection. 5 September 2008. http://www.davidrumsey.com

1880

Letts, Son & Co. 1883. The Environs of Paris [map]. David Rumsey Map Collection. 5 September 2008. http://www.davidrumsey.com

1900

Evenson, Norma. 1979. Paris: A Century of Change: 1878 – 1978. New Haven: Yale University Press, p. 328.

1928

Touring Club Italiano. 1977. Atlante Internazionale del Touring Club Italiano. Milano: Touring Club Italiano.

1955

US Army Map Service. 1955. Paris, Sheet NM 31-11 Series M562 [map]. Perry-Castañeda Library Map Collection. 4 September 2008. University of Texas Libraries. http://www.lib.utexas.edu/maps/

US Army Map Service. 1955. Paris, Sheet NM 31-8 Series M562 [map]. Perry-Castañeda Library Map Collection. 4 September 2008. University of Texas Libraries. http://www.lib.utexas.edu/maps/

1974

Rand McNally and Company. 1987. Rand McNally Cosmopolitan World Atlas. Chicago: Rand McNally.

**Santiago**

1800 - 1940

Correa, Pastor P., et al. 2002. Retrospectiva de un Ensayo de Planificación del Gran Santiago 1952. Santiago: Universidad Central de Chile, Facultad de Arquitectura y Bellas Artes, p. 51.

1940 – 1970

Galetovic, Alexander, ed. 2006. Santiago: Dónde Estamos y Hacia Dónde Vamos. Santigo de Chile: Centro de Estudios Públicos, front cover.

**Shanghai**

1810

Johnson, Linda C. 1995. Shanghai: From market Town to Treaty Port, 1074 – 1858. Stanford: Stanford University Press, p. 114.

1853

Goodman, Byrna. 1995. Native Place, City and Nation: Regional Networks and Identities in Shanghai 1853 – 1937. Berkeley: University of California Press, p. 52

1875

Yuchang, Xu and Zuoru, Feng. 1875. Shanghai xian chengxiang zujie quan tu (上海縣城廂租界全圖) [map]. Virtual Shanghai: Shanghai Urban Space in Time. 8 Sept. 2008. http://virtualshanghai.ish-lyon.cnrs.fr/

1902

Capitaine Gadoffre. 1902. Chang-hai et environs [map]. Virtual Shanghai: Shanghai Urban Space in Time. 8 Sept. 2008. http://virtualshanghai.ish-lyon.cnrs.fr/

1914

Naval School of Military Administration. 1914. City of Shanghai (China) and surrounding region - Base map - Urban built-up areas and military barracks [map]. Virtual Shanghai: Shanghai Urban Space in Time. 8 Sept. 2008. http://virtualshanghai.ish-lyon.cnrs.fr/

1944

US Army Map Service. 1944. Shanghai (Eastern China) [map]. Perry-Castañeda Library Map Collection. 8 September 2008. University of Texas Libraries. http://www.lib.utexas.edu/maps/

1973

Author unkown. 1973. Shanghai (in Russian) [map]. Virtual Shanghai: Shanghai Urban Space in Time. 8 Sept. 2008. http://virtualshanghai.ish-lyon.cnrs.fr/

**Sydney**

1808

Lesueur, Charles A. 1808. Plan von Sydney der Hauptstadt der Englischen Colonien in Australien [map]. 28 Aug. 2008. National Library of Australia, Digital Collections - Maps. http://www.nla.gov.au/digicoll/maps.html

1833

Society for the Diffusion of Useful Knowledge. 1833. New South Wales, Sydney [map]. David Rumsey Map Collection. 8 September 2008. http://www.davidrumsey.com

1860

Stones, William. Australia 1860? [map] in: “My First Voyage: A Book for Youth.” Second Edition. London: Simpkin, Marshall & Co. 1860. Perry-Castañeda Library Map Collection. 4 September 2008. University of Texas Libraries. http://www.lib.utexas.edu/maps/

1883

Letts, Son & Co. 1883. New South Wales [map]. David Rumsey Map Collection. 8 September 2008. http://www.davidrumsey.com

1895

Appleton, D & Co. 1890-1899. Sydney and Environs [map]. 28 Aug. 2008. National Library of Australia, Digital Collections - Maps. http://www.nla.gov.au/digicoll/maps.html

1917, 1945, 1975

New South Wales Government. History of Sydney’s Growth [map]. In: “City of Cities: A Plan for Sydney’s Future,” Department of Planning, New South Wales, 2005. Sydney Metropolitan Strategy, New South Wales Government. http://www.metrostrategy.nsw.gov.au/dev/

**Warsaw**

1794

Schropp, Simon & Co. 1794. Gegend der Stadt Warschau mit allen umliegenden Oertern [map]. Nov. 15 2008. ftp://mapy.ziomal.org/mapy/plany_miast/Warszawa/

1831

Society for the Diffusion of Useful Knowledge. 1831. Warsaw (Warszawa) [map]. David Rumsey Map Collection. 5 September 2008. http://www.davidrumsey.com

1867

Author unknown. 1867. Bapwaba Warszawa [map]. Nov. 15 2008. ftp://mapy.ziomal.org/mapy/plany_miast/Warszawa/

1888

Meyer, Herman J. 1892. Meyers grosse konversations-lexicon 4th Edition, Volume 16. Wien und Leipzig: Bibliographisches Institut, p. 400.

1915

Author Unknown. 1915. Warsaw? [map]. Nov. 15 2008. ftp://mapy.ziomal.org/mapy/plany_miast/Warszawa/WAWA_INZ1/

1936

Origin unknown, reference missing

1958

Origin unknown, reference missing

1978

Panstwowe Przedsiebiorstwo Wydawnictw Cartograficznych. 1978. Warszawa Plan Miasta [map]. Nov. 15 2008. http://www.trasbus.com/plan1978-1979.htm
