# Supplementary material for: The Relationship between Habitat Loss and Fragmentation during Urbanization: An Empirical Evaluation from 16 World Cities
Source: PLoS One. 2016 Apr 28;11(4):e0154613. doi: 10.1371/journal.pone.0154613 (PMC4849762; doi:10.1371/journal.pone.0154613)
Supplement: S3 Appendix — (DOC) [file pone.0154613.s003.doc]

**S3 Appendix. Verification of data consistency and methods.**

**
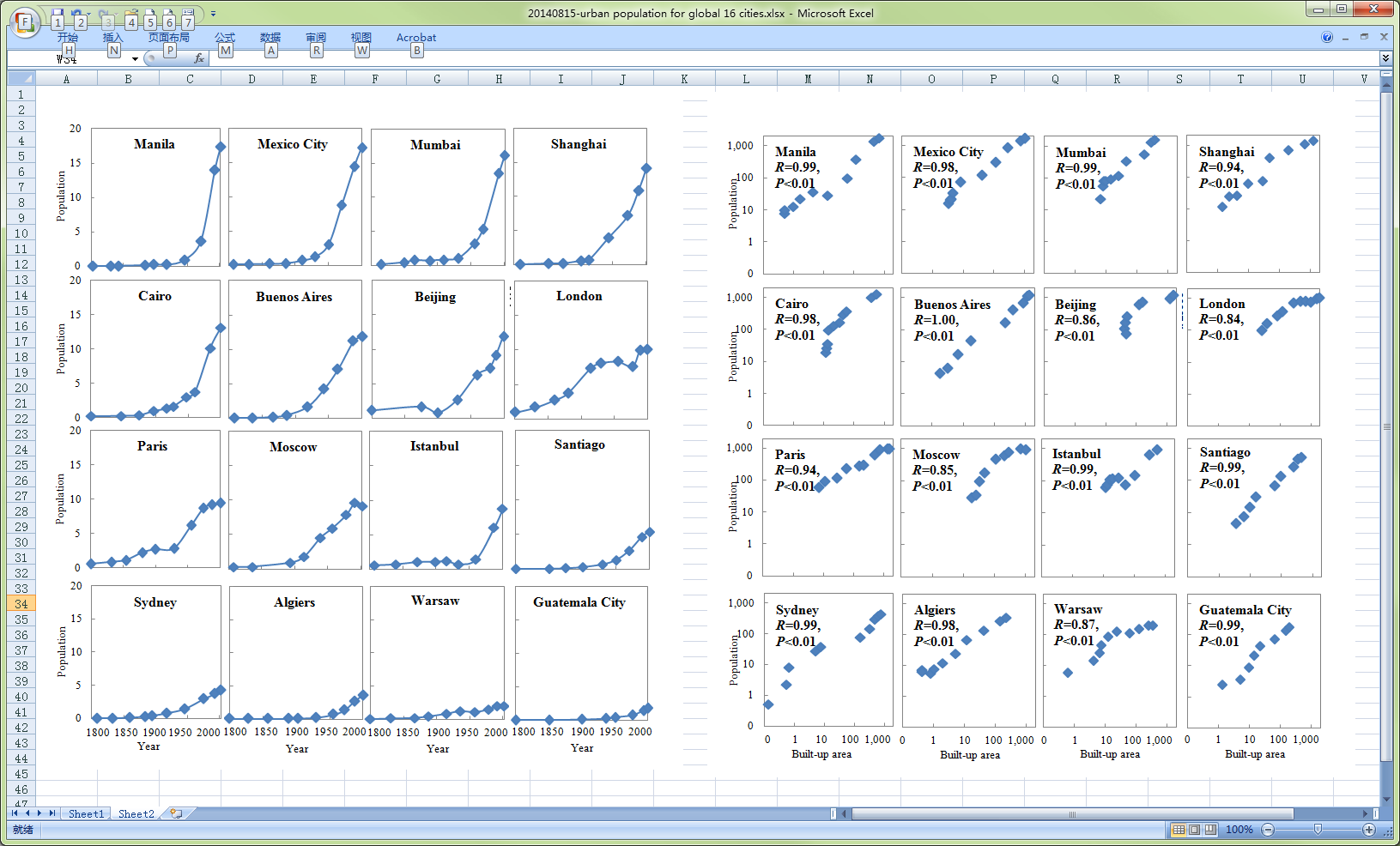
**

Figure A. Changes in urban population from 1800 to 2000 (million people).

**
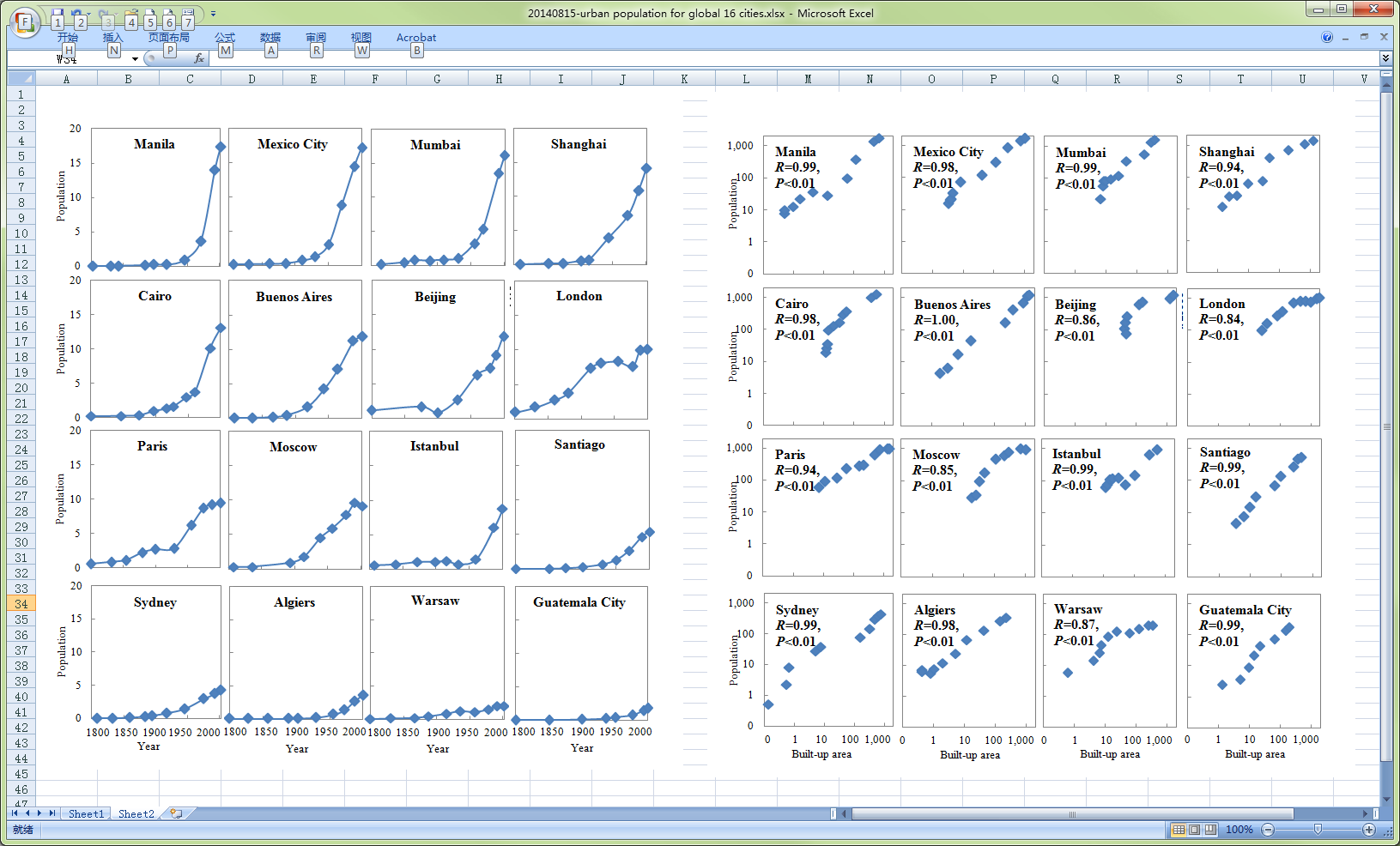
**

Figure B. Correlation between built-up area (in km^2^) and urban population (10 thousand people) during 1800-2000.


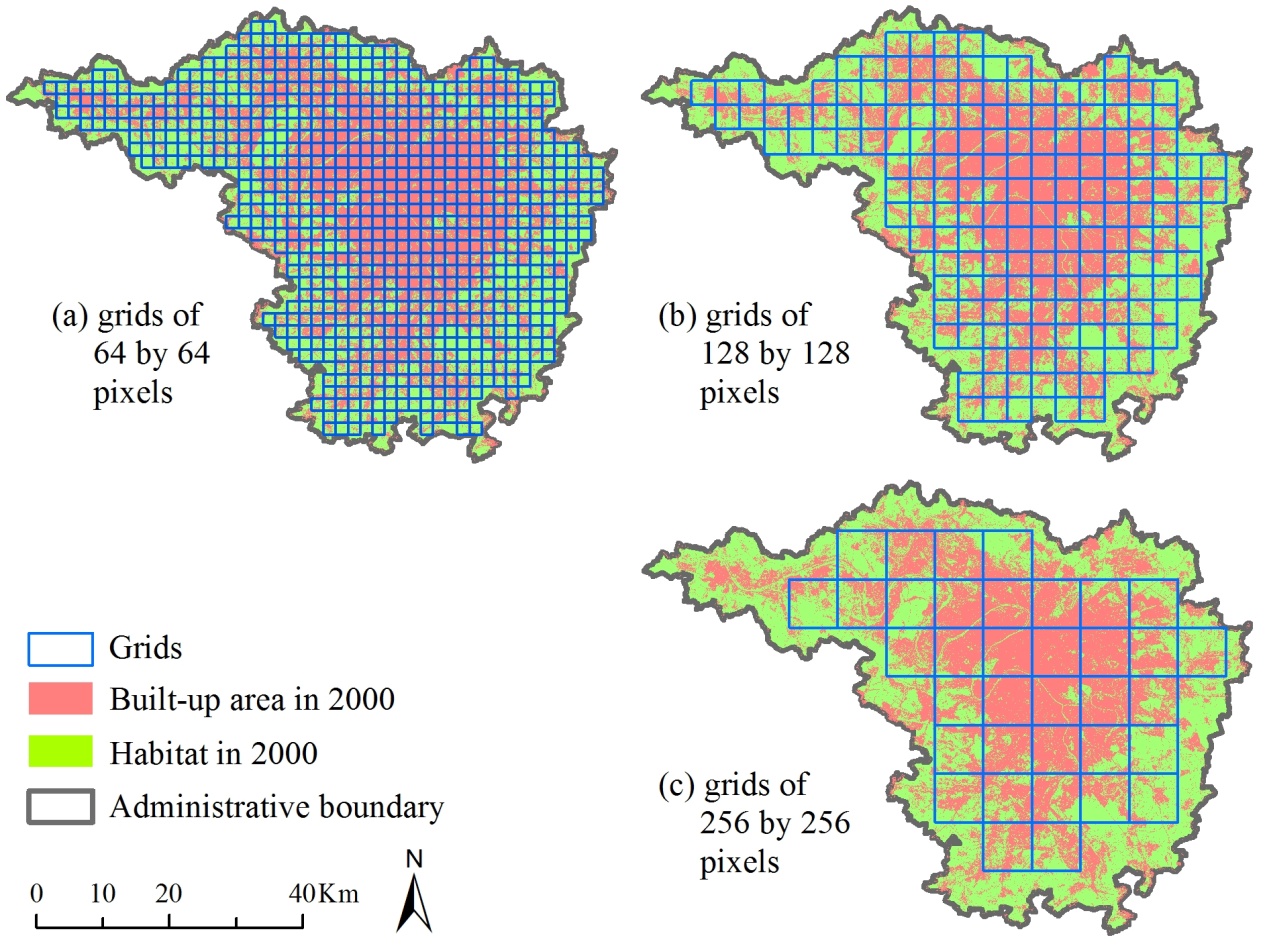


Figure C. Grids with different spatial extents, including 64 by 64 pixels (a), 128 by 128 pixels (b), and 256 by 256 pixels (c), used for space-for-time analysis in Paris in 2000.
