# Supplementary material for: The Relationship between Habitat Loss and Fragmentation during Urbanization: An Empirical Evaluation from 16 World Cities
Source: PLoS One. 2016 Apr 28;11(4):e0154613. doi: 10.1371/journal.pone.0154613 (PMC4849762; doi:10.1371/journal.pone.0154613)
Supplement: S4 Appendix — (DOC) [file pone.0154613.s004.doc]

**S4 Appendix. The relationships between habitat loss and habitat fragmentation during urbanization based on historical urbanization data and space-for-time analysis.**


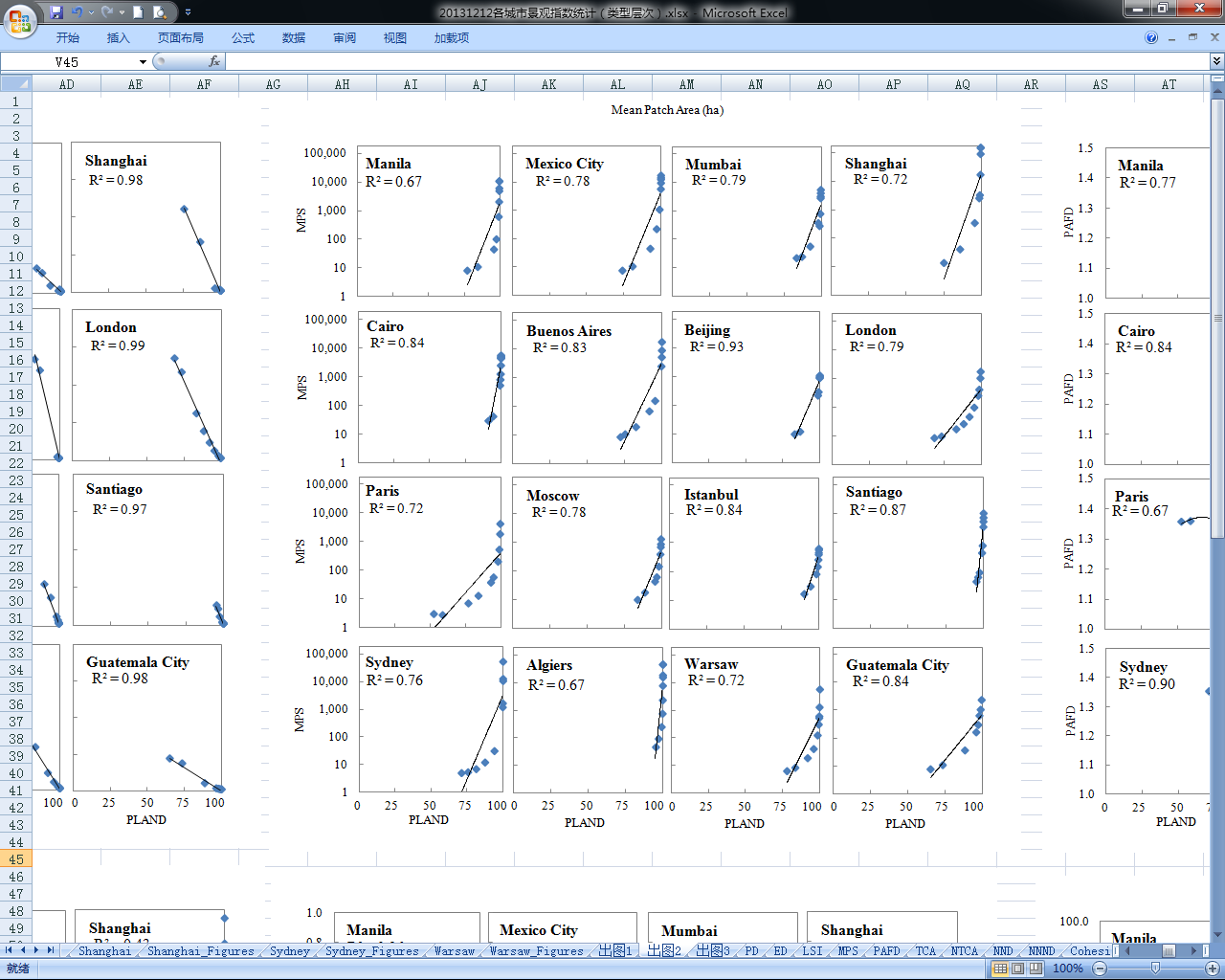


**(a)**


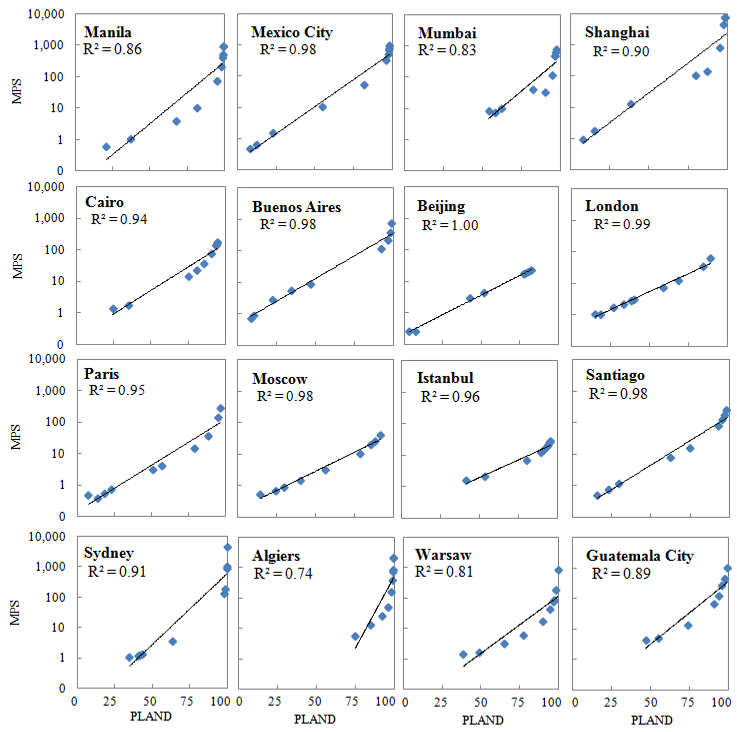


**(b)**

Figure A. The relationship between percentage of habitat (PLAND) and mean patch size (MPS; in ha) of habitat at the urban regional extent (a) and at the central city area extent (b).


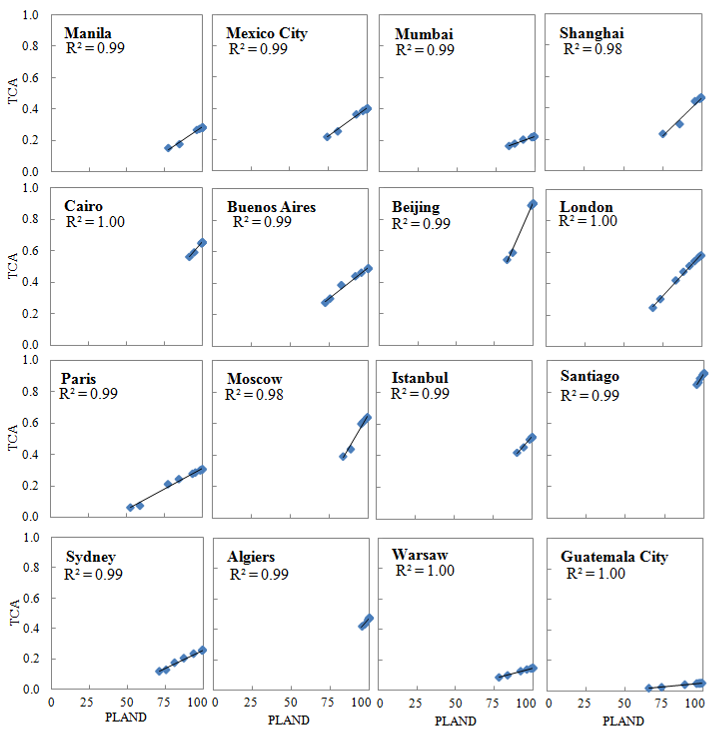


**(a)**

**(b)**


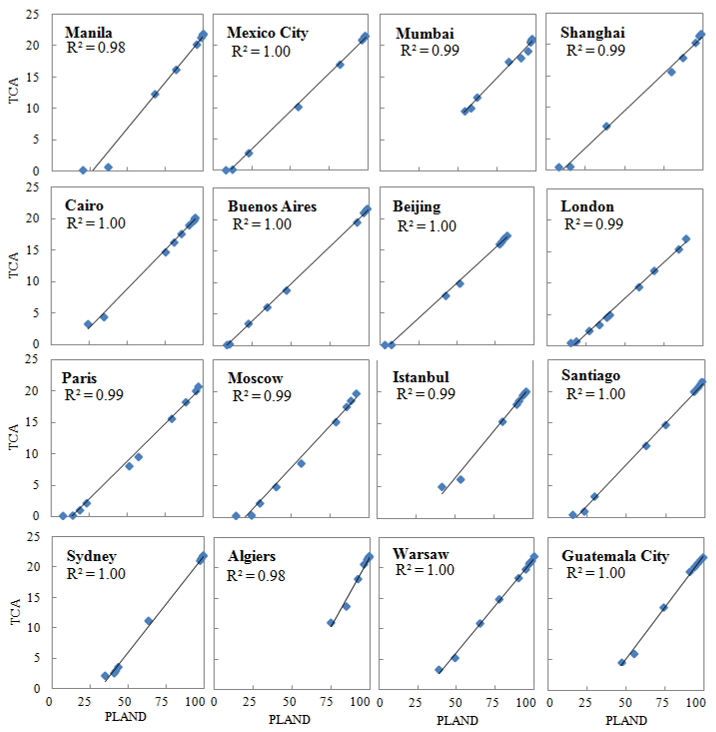


Figure B. The relationship between percentage of habitat (PLAND) and total core area (TCA; in ha) of habitat at the urban regional extent (a) and at the central city area extent (b).


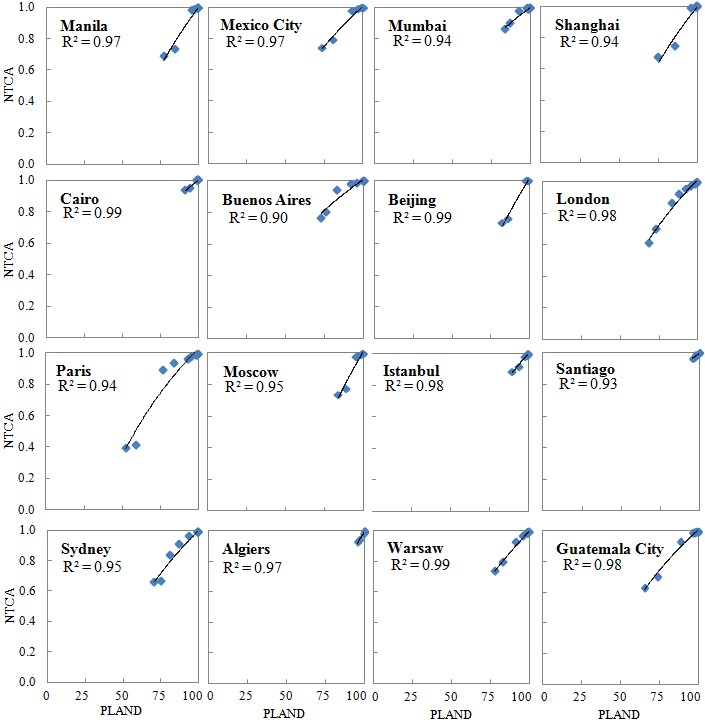


**(a)**

**(b)**


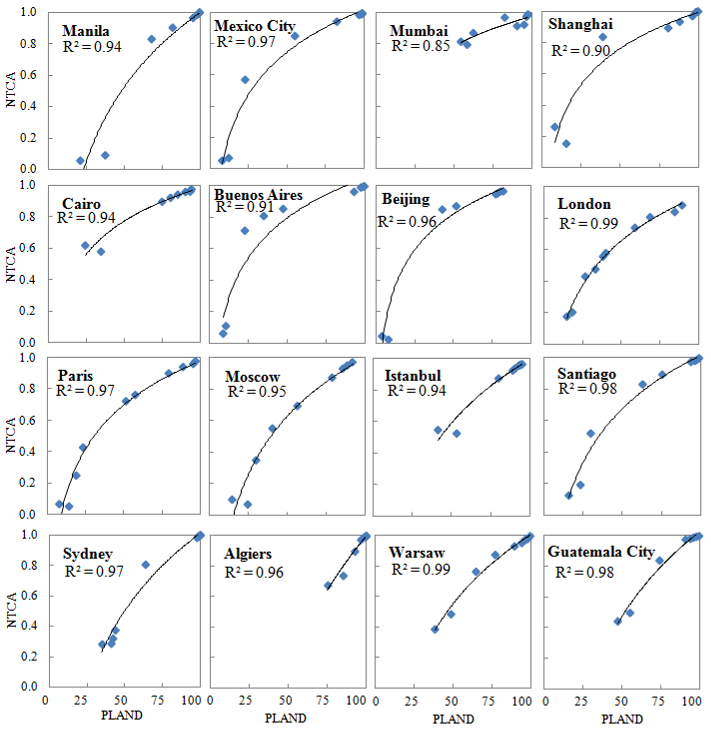


Figure C. The relationship between percentage of habitat (PLAND) and normalized total core area (NTCA) of habitat at the urban regional extent (a) and the central city area extent (b).


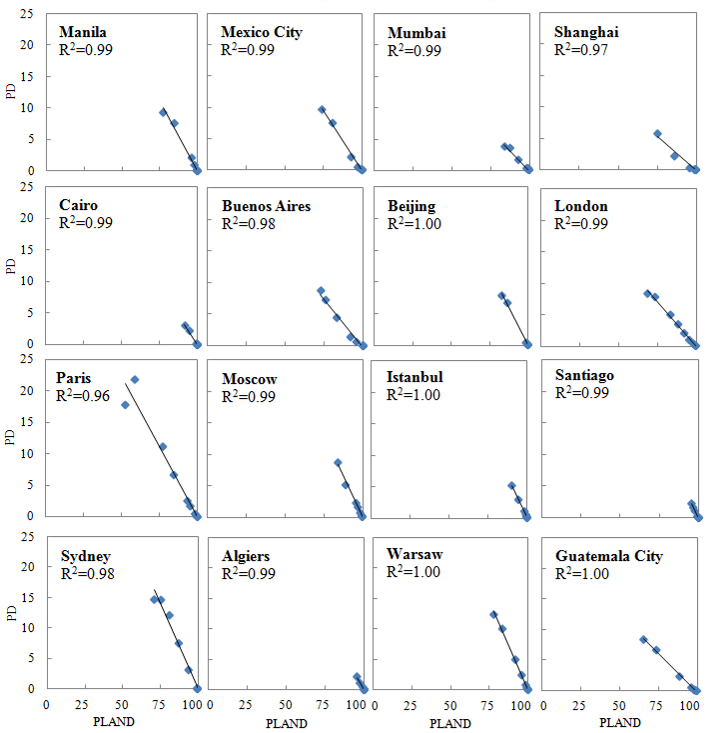


**(a)**

**(b)**


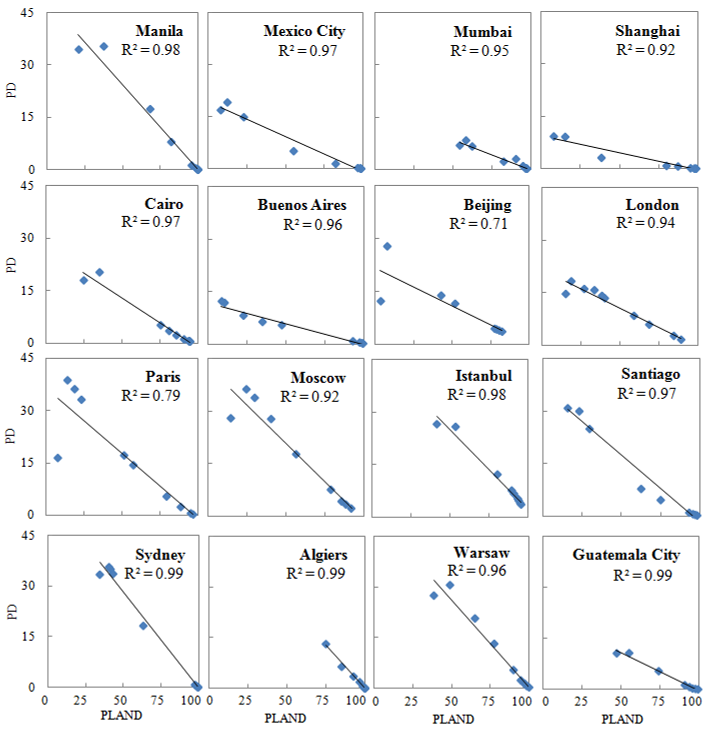


Figure D. The relationship between percentage of habitat (PLAND) and patch density (PD; the meter per ha) of habitat at the urban regional extent (a) and at the central city area extent (b).


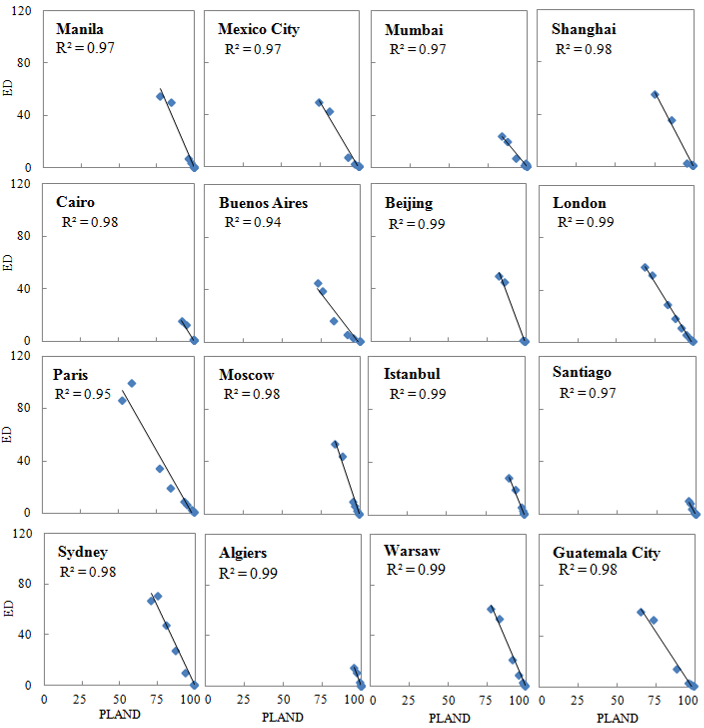


**(a)**

**(b)**


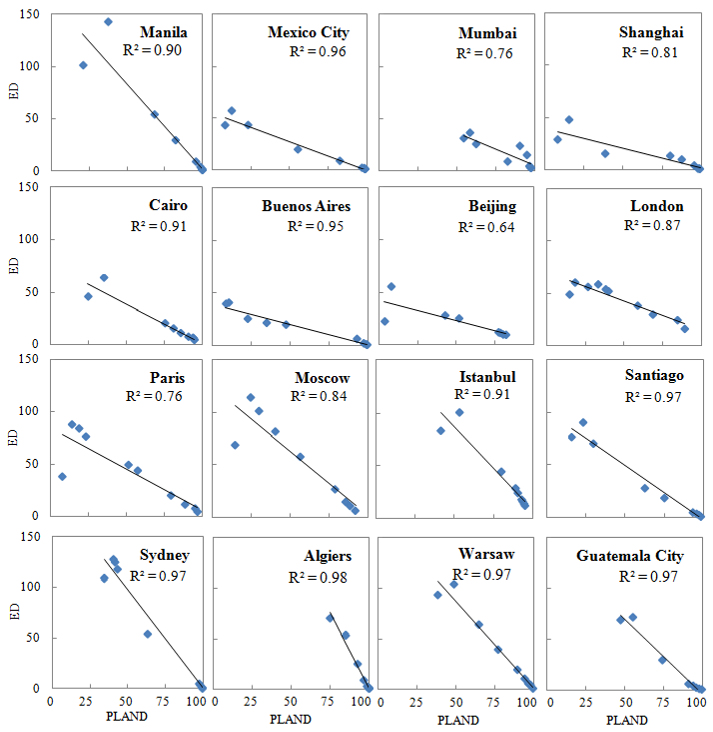


Figure E. The relationship between percentage of habitat (PLAND) and edge density (ED; the meter per ha) of habitat at the urban regional extent (a) and at the central city area extent (b).


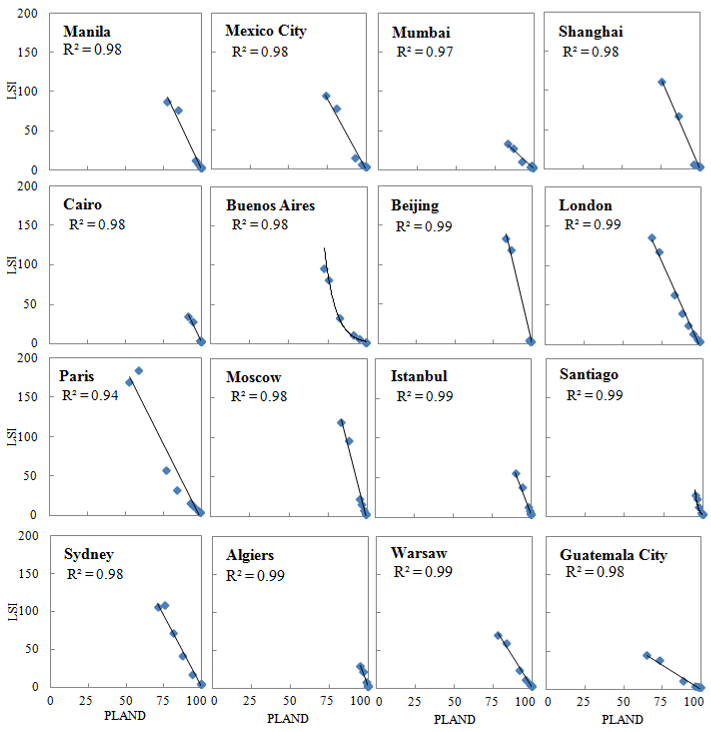


**(a)**

**(b)**


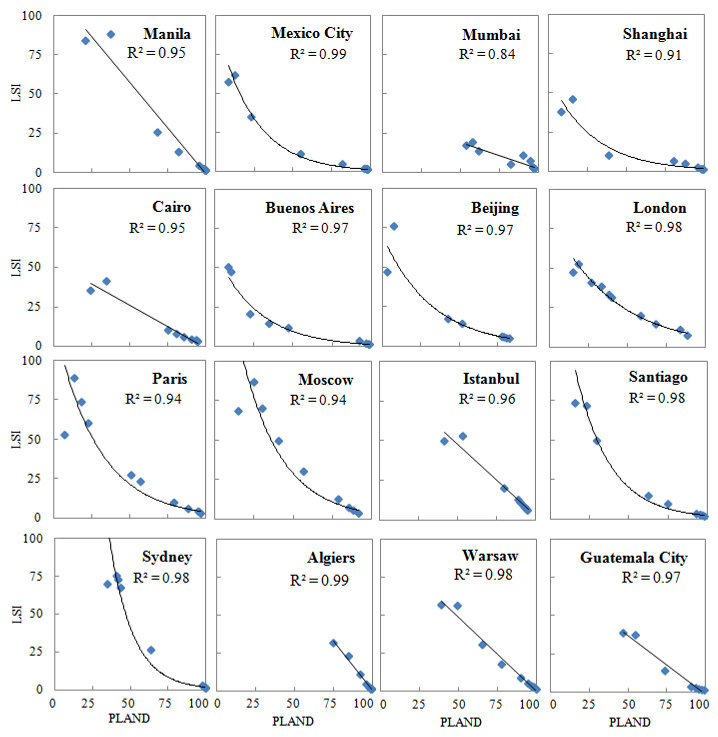


Figure F. The relationship between percentage of habitat (PLAND) and landscape shape index (LSI) of habitat at the urban regional extent (a) and the central city area extent (b).


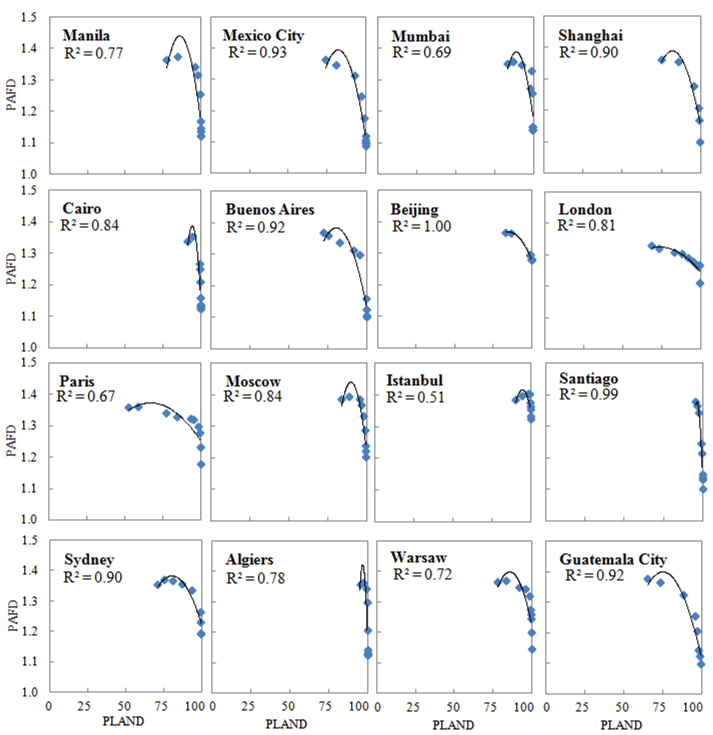


**(a)**

**(b)**


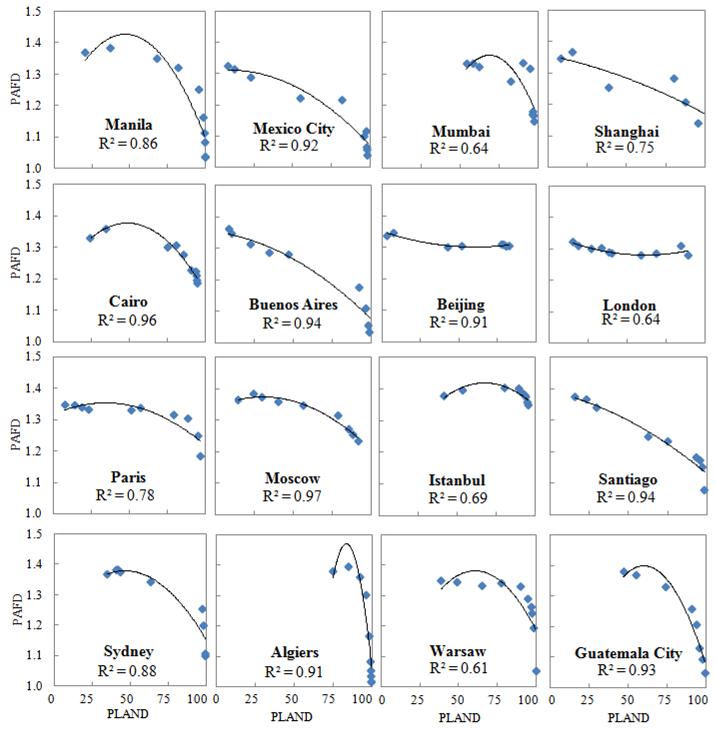


Figure G. The relationship between percentage of habitat (PLAND) and perimeter-area fractal dimension (PAFD) of habitat at the urban regional extent (a) and at the central city area extent (b).


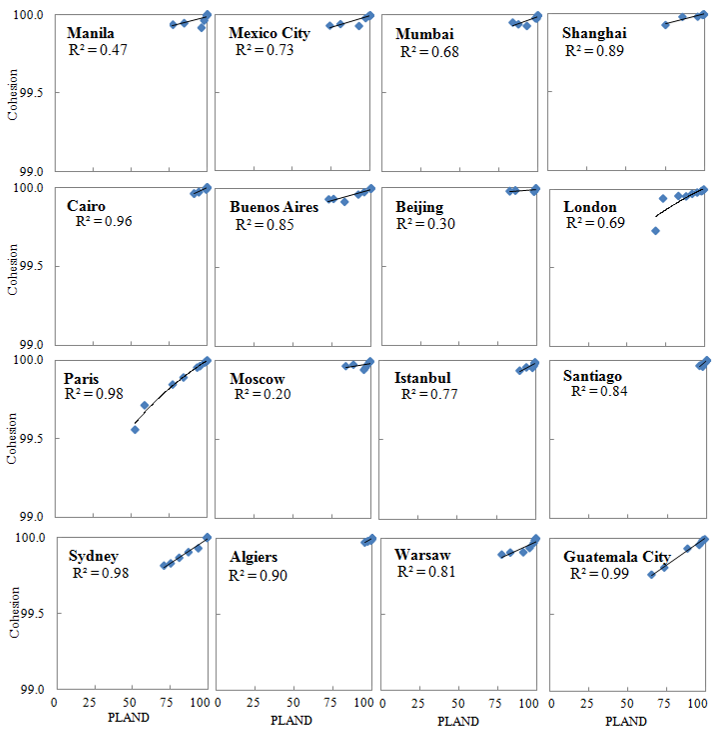


**(a)**

**(b)**


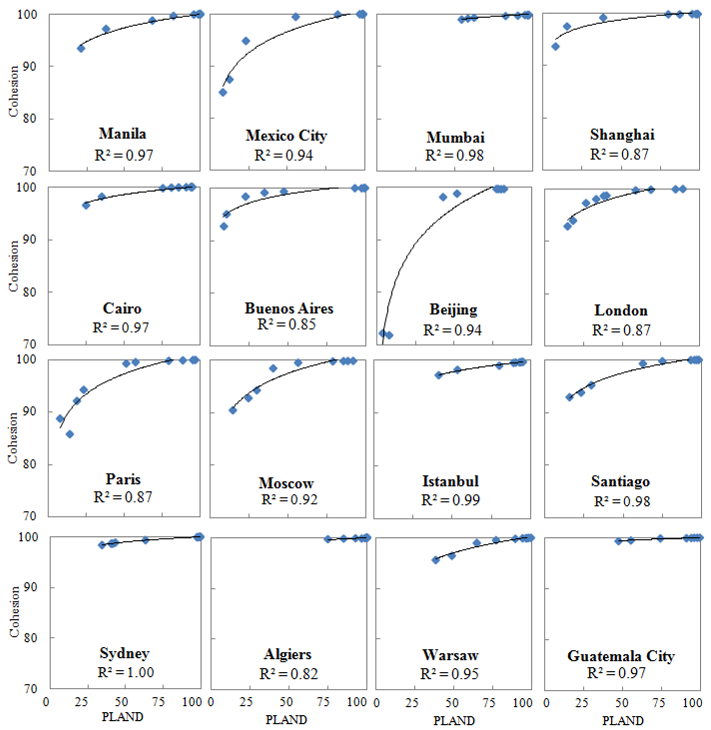


Figure H. The relationship between percentage of habitat (PLAND) and Cohesion of habitat at the urban regional extent (a) and at the central city area extent (b).


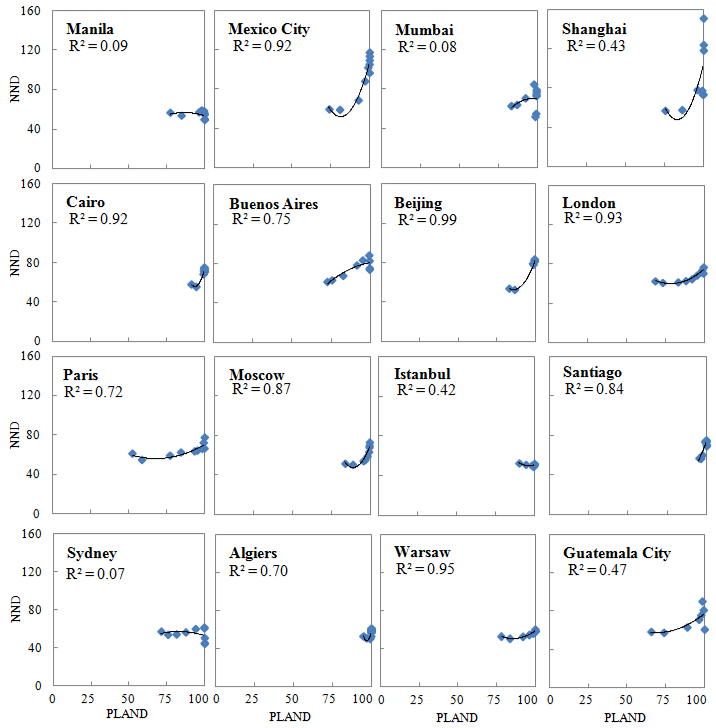


**(a)**

**(b)**


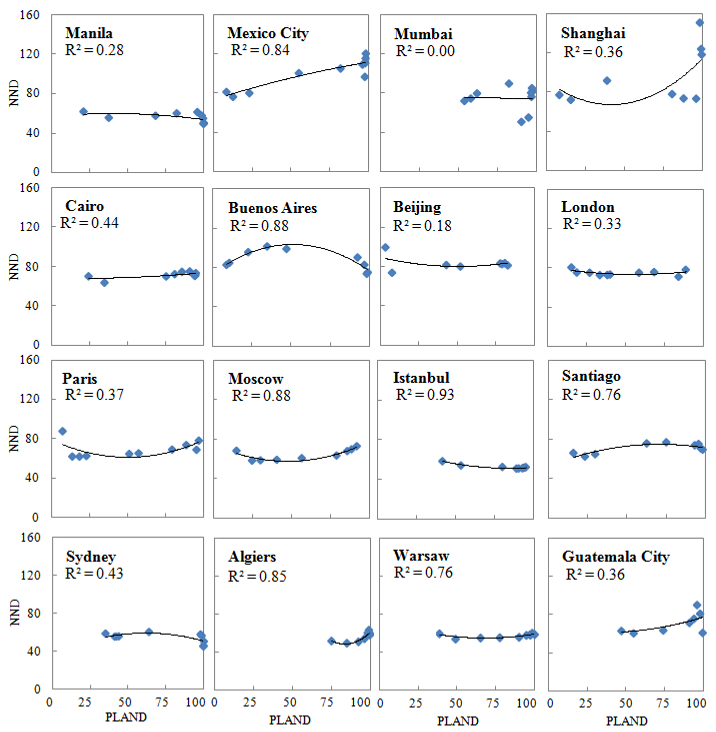


Figure I. The relationship between percentage of habitat (PLAND) and nearest neighbor distance (NND; in meter) of habitat at the urban regional extent (a) and at the central city area extent (b).


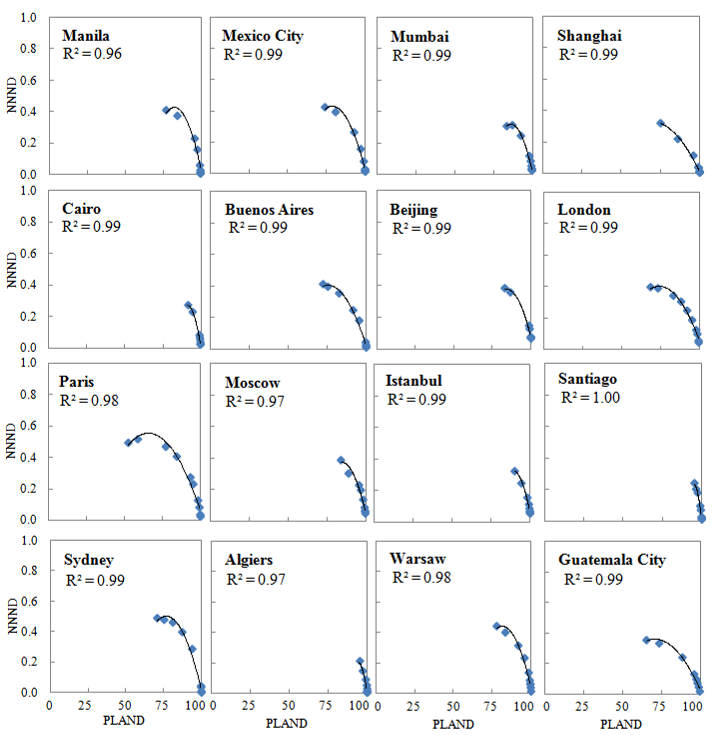


**(a)**

**(b)**


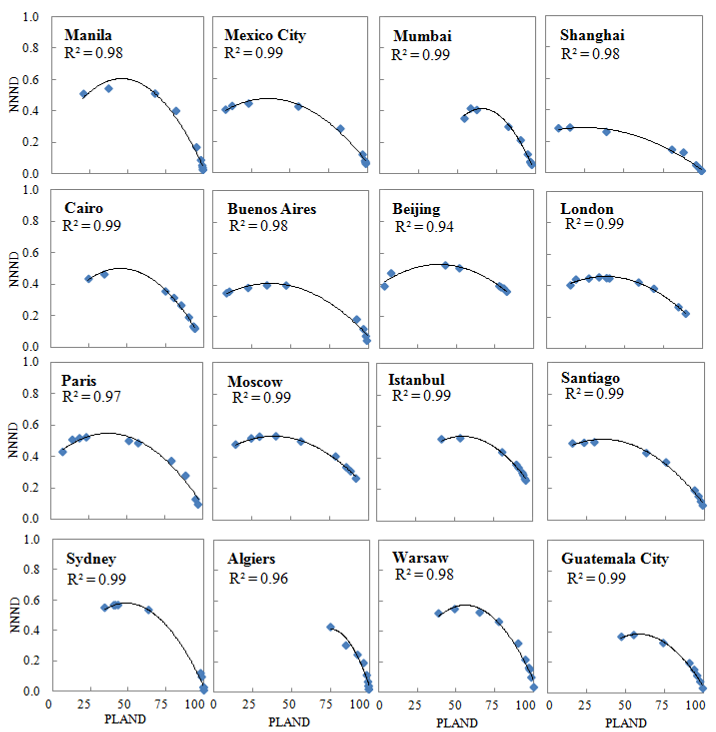


Figure J. The relationship between percentage of habitat (PLAND) and normalized nearest neighbor distance (NNND) of habitat at the urban regional extent (a) and at the central city area extent (b).


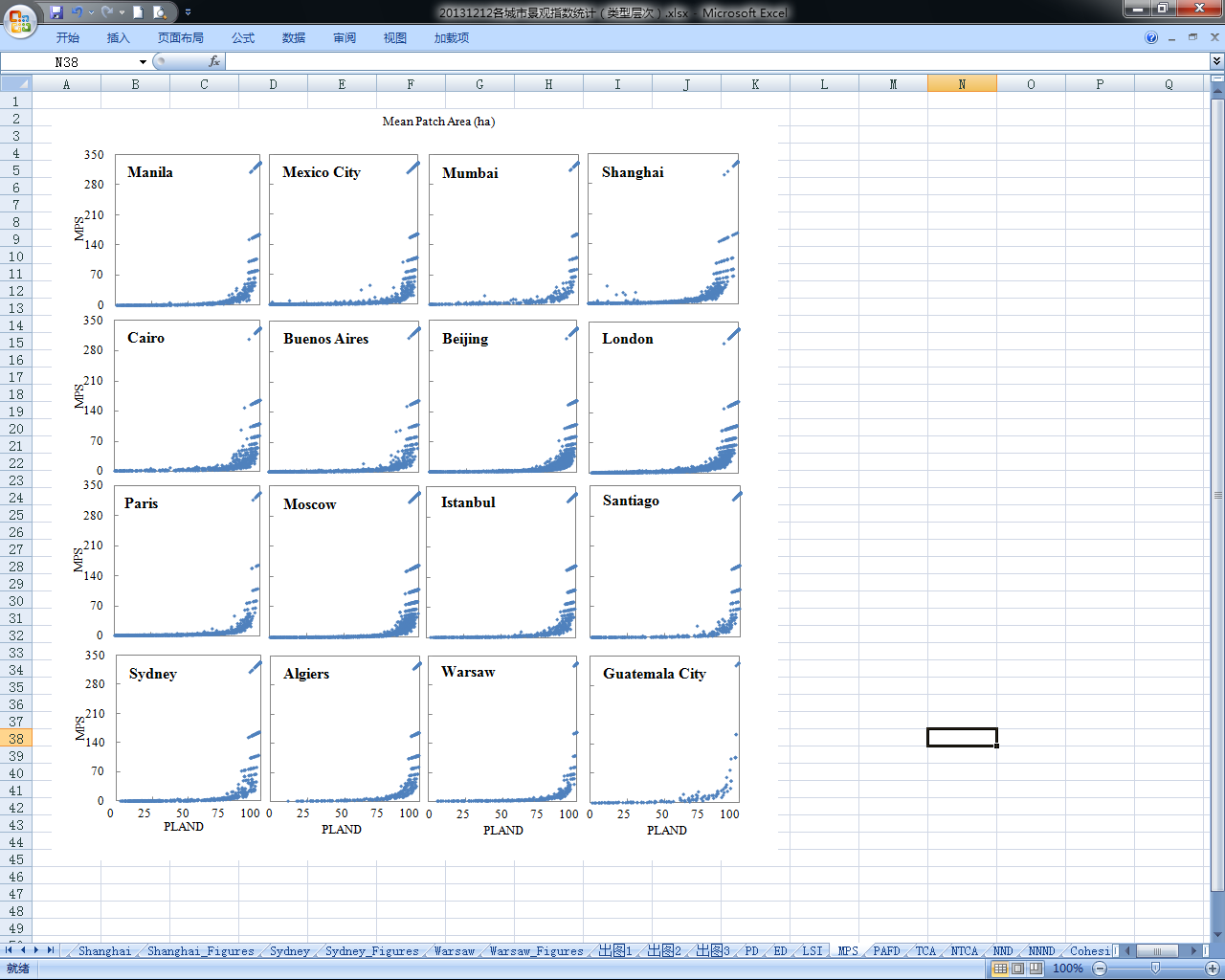


Figure K. The relationship between percentage of habitat (PLAND) and mean patch size (MPS; in ha) of habitat based on space-for-time analysis in 2000 at the extent of 64 by 64 pixels.


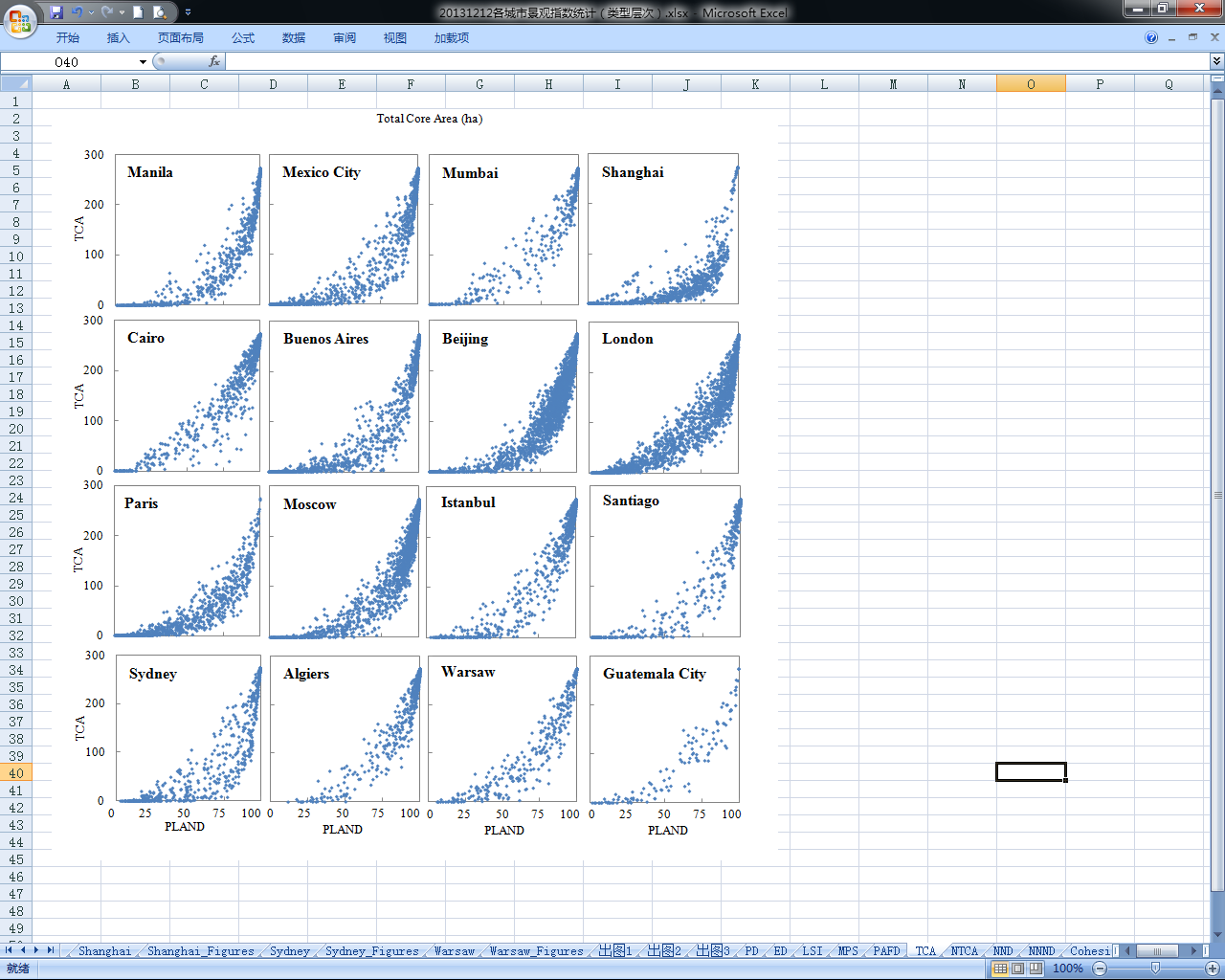


Figure L. The relationship between percentage of habitat (PLAND) and total core area (TCA; in ha) of habitat based on space-for-time analysis in 2000 at the extent of 64 by 64 pixels.


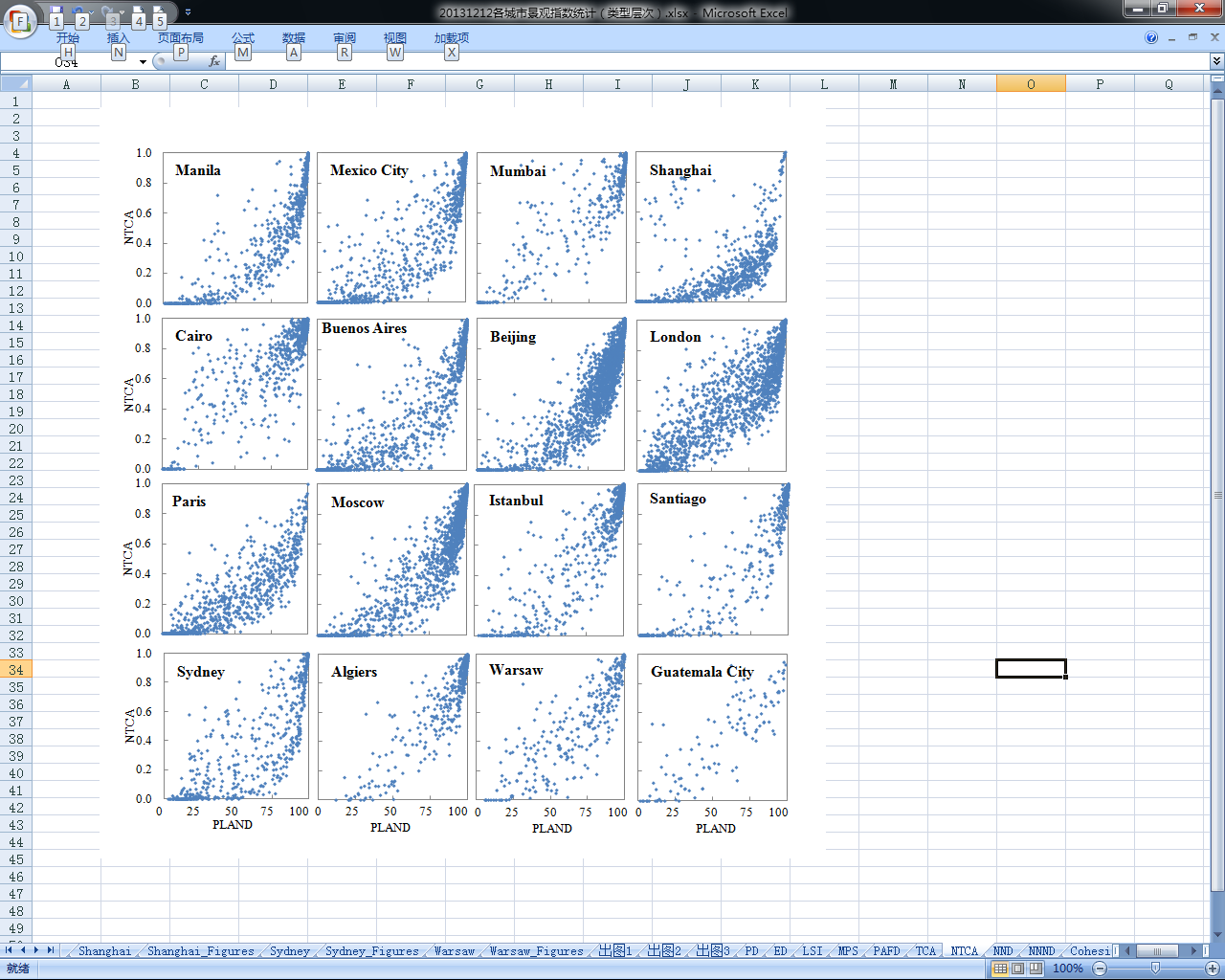


Figure M. The relationship between percentage of habitat (PLAND) and normalized total core area (NTCA) of habitat based on space-for-time analysis in 2000 at the extent of 64 by 64 pixels.


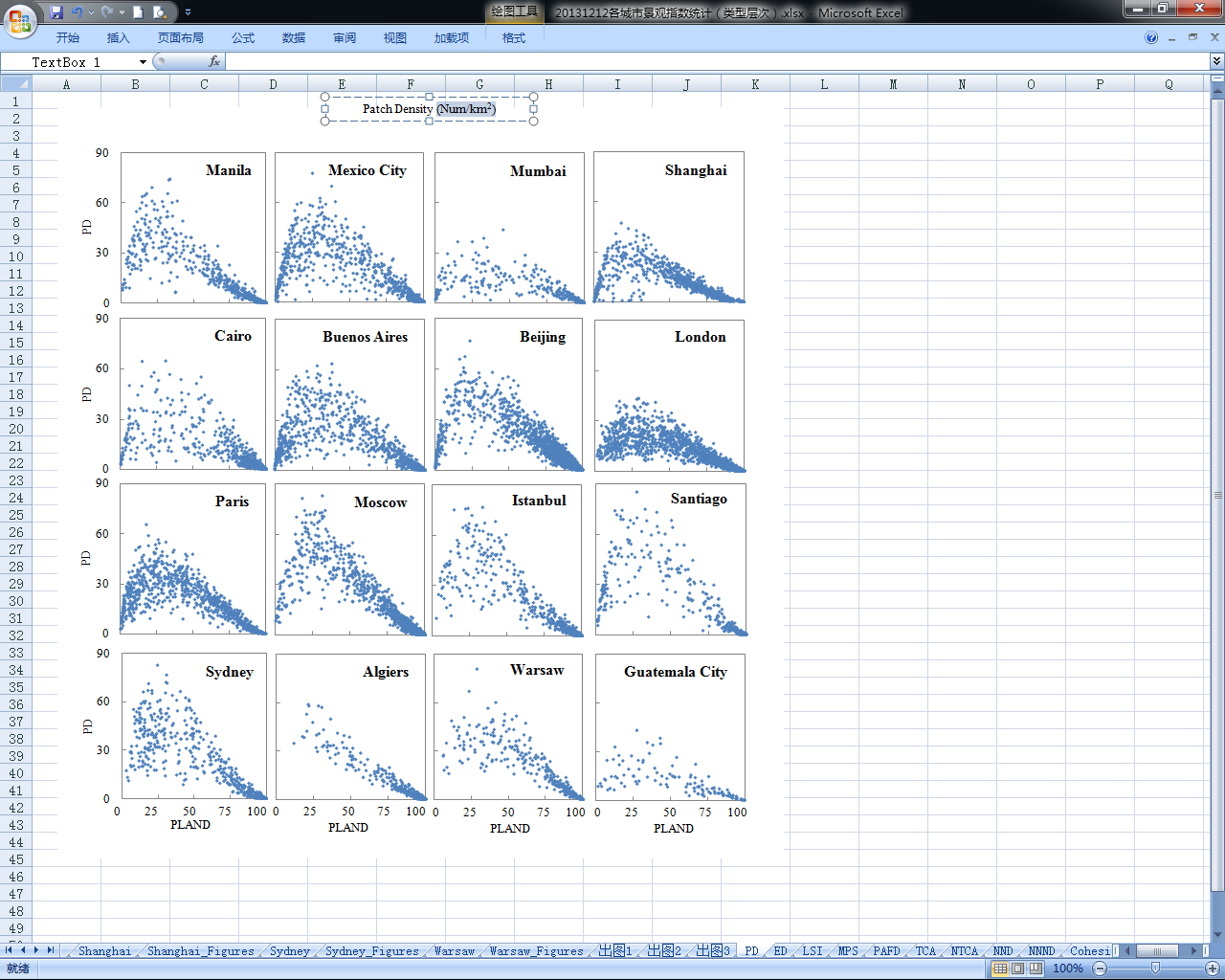


Figure N. The relationship between percentage of habitat (PLAND) and patch density (PD; the meter per ha) of habitat based on space-for-time analysis in 2000 at the extent of 64 by 64 pixels.


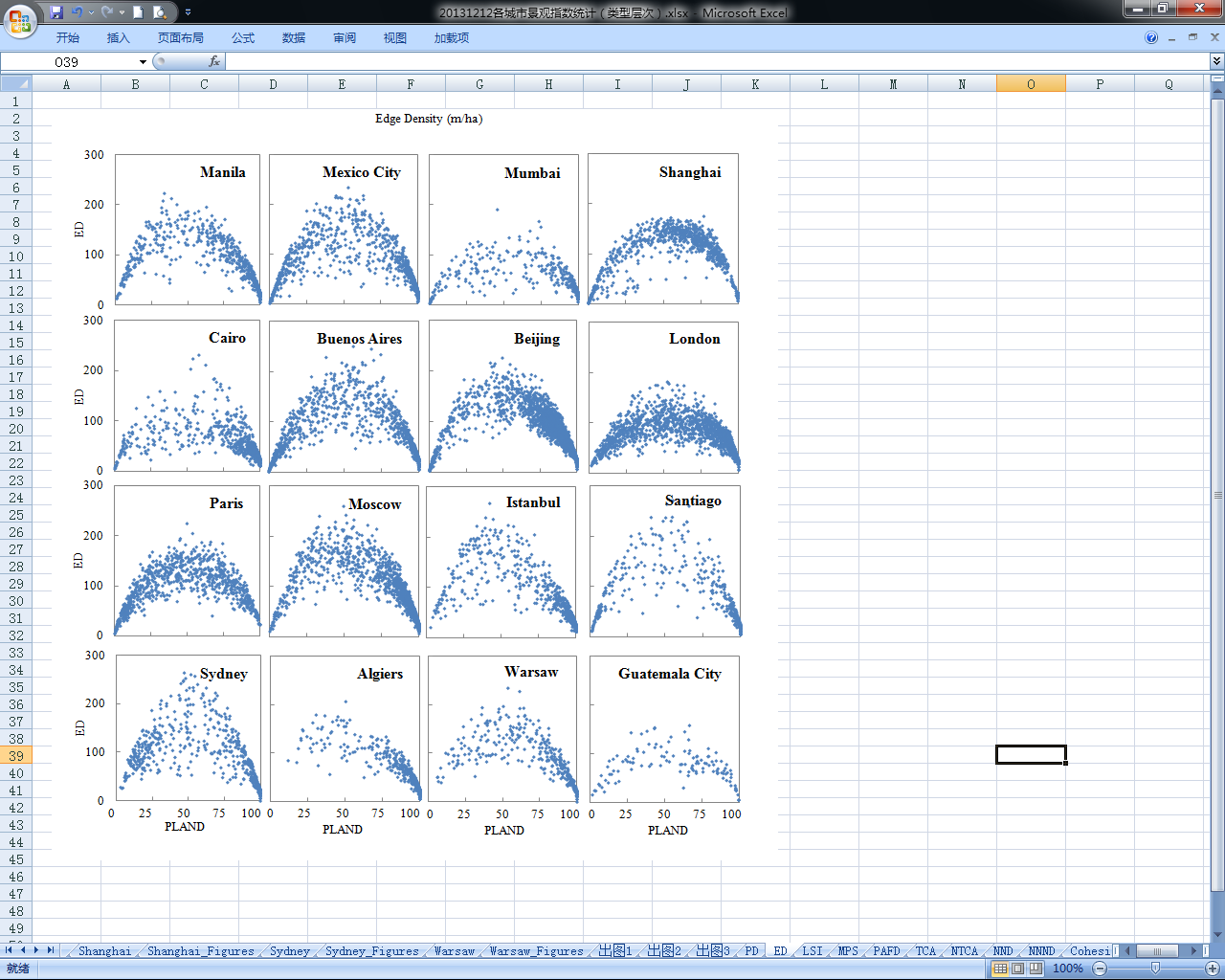


Figure O. The relationship between percentage of habitat (PLAND) and edge density (ED; the meter per ha) of habitat based on space-for-time analysis in 2000 at the extent of 64 by 64 pixels.


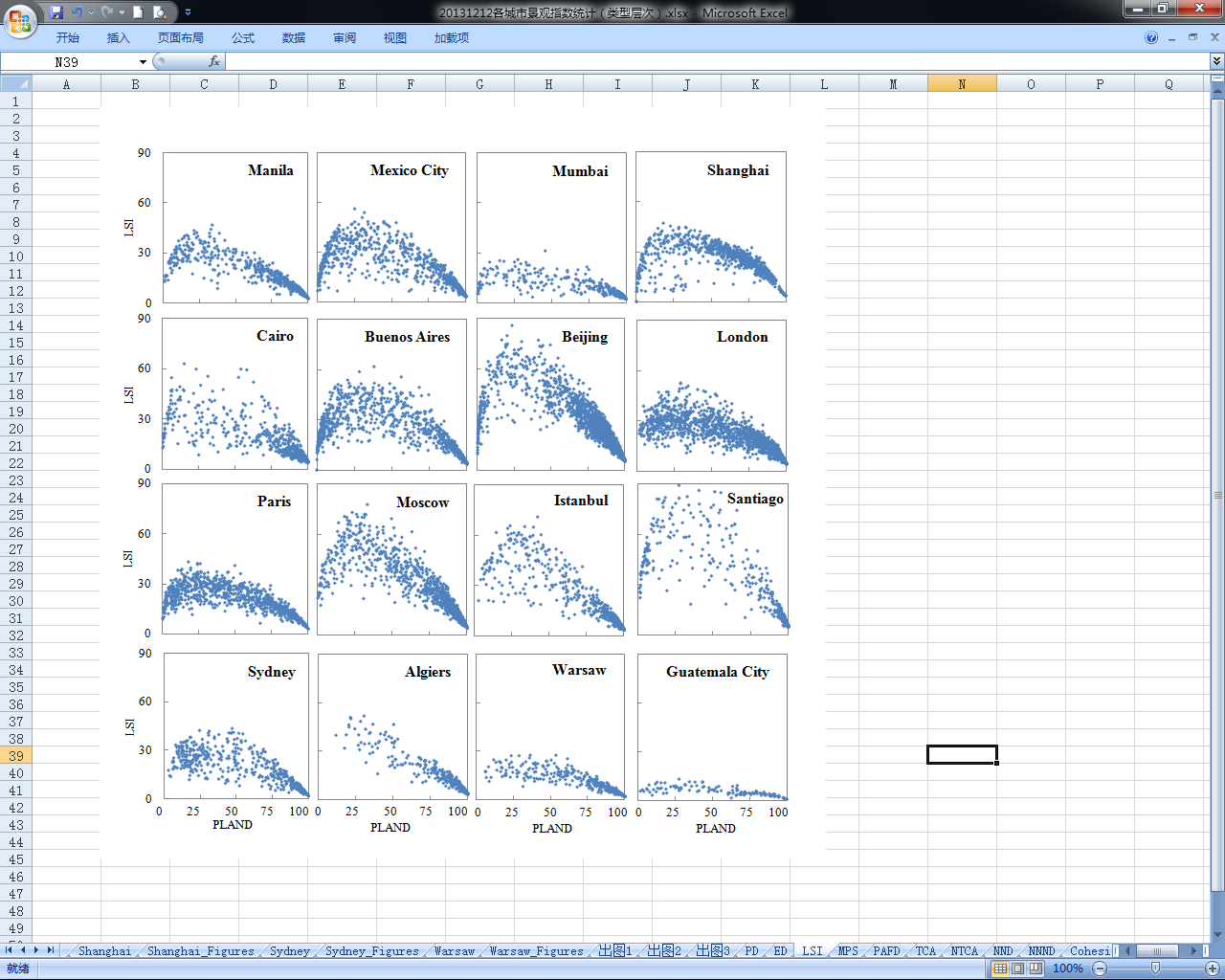


Figure P. The relationship between percentage of habitat (PLAND) and landscape shape index (LSI) of habitat based on space-for-time analysis in 2000 at the extent of 64 by 64 pixels.


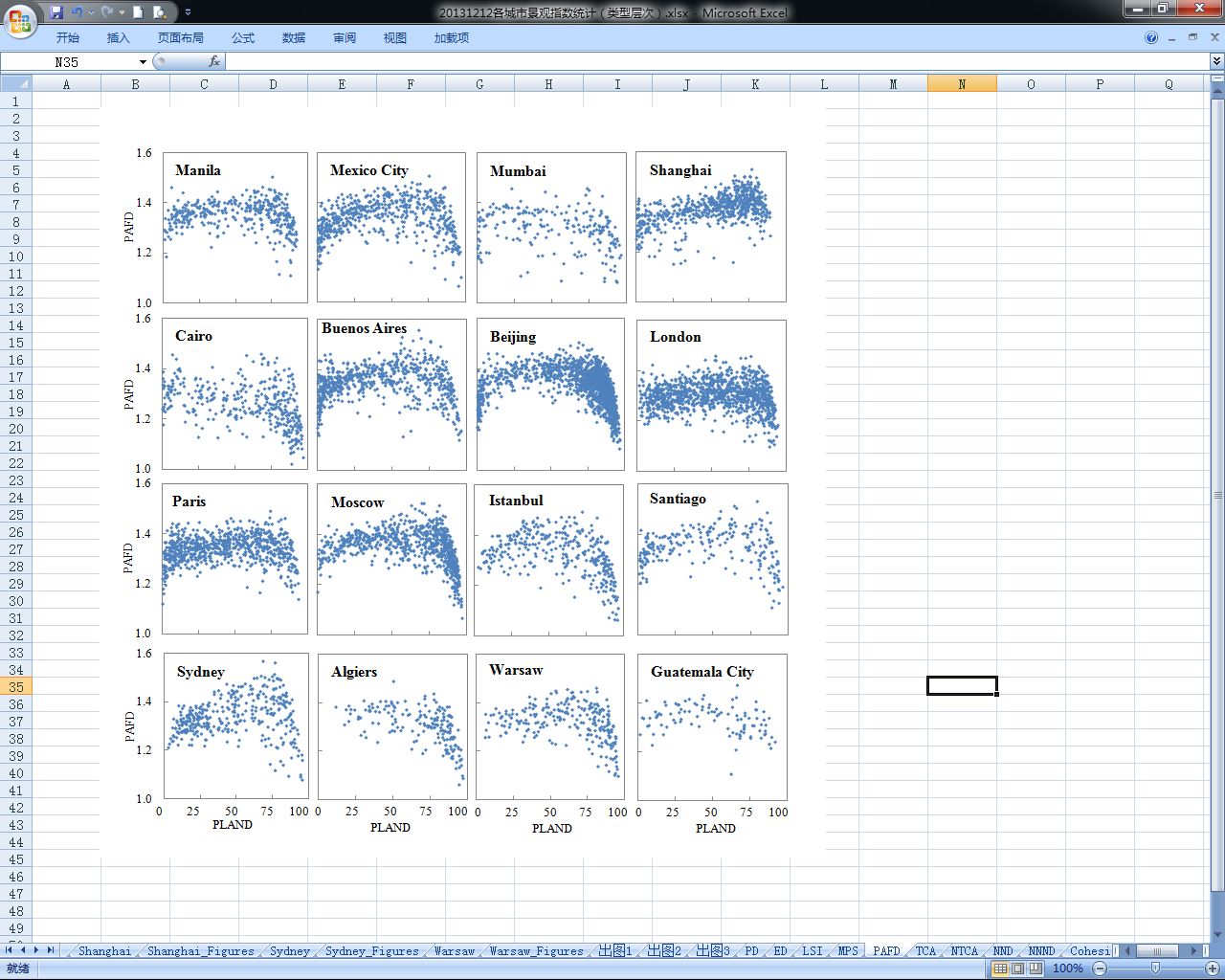


Figure Q. The relationship between percentage of habitat (PLAND) and perimeter-area fractal dimension (PAFD) of habitat based on space-for-time analysis in 2000 at the extent of 64 by 64 pixels.


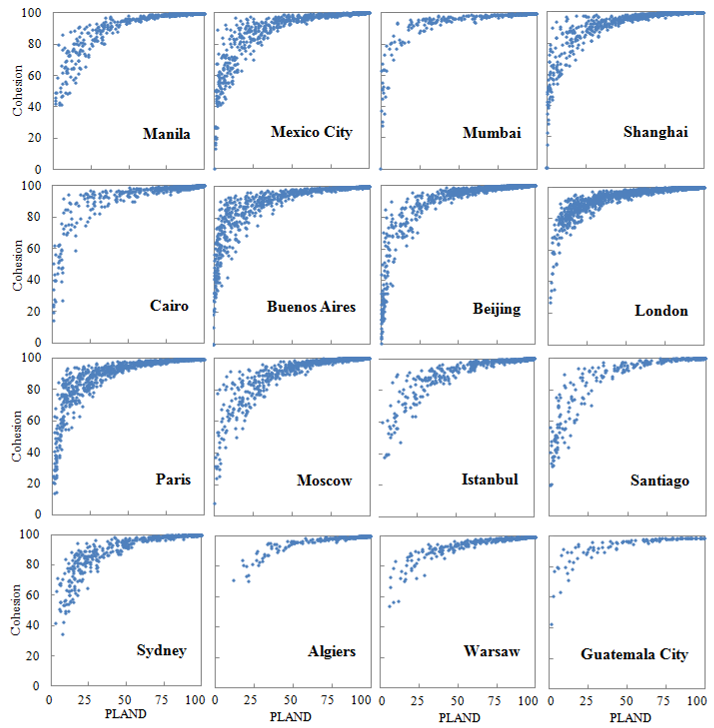


Figure R. The relationship between percentage of habitat (PLAND) and Cohesion of habitat based on space-for-time analysis in 2000 at the extent of 64 by 64 pixels.


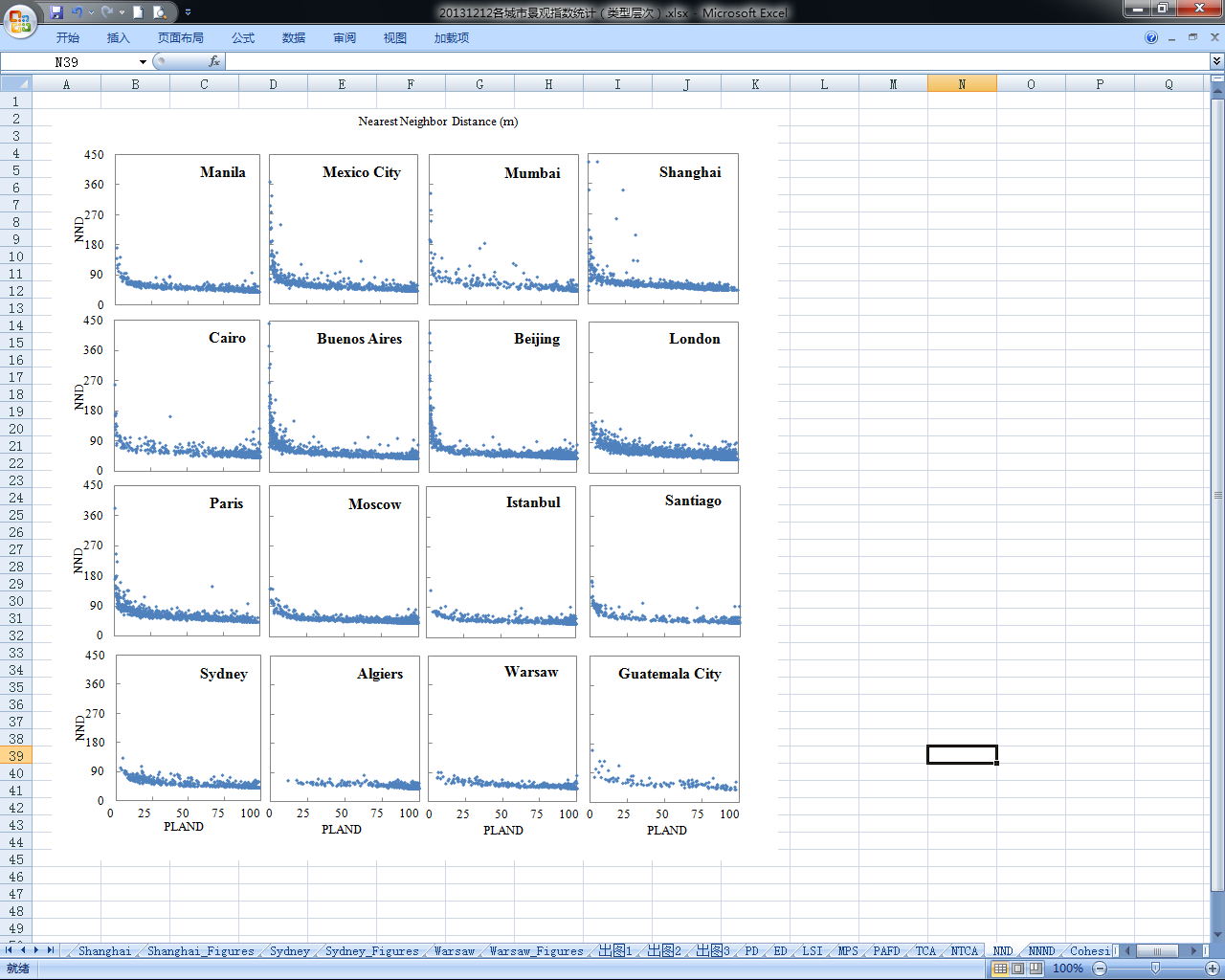


Figure S. The relationship between percentage of habitat (PLAND) and nearest neighbor distance (NND; in meter) of habitat based on space-for-time analysis in 2000 at the extent of 64 by 64 pixels.


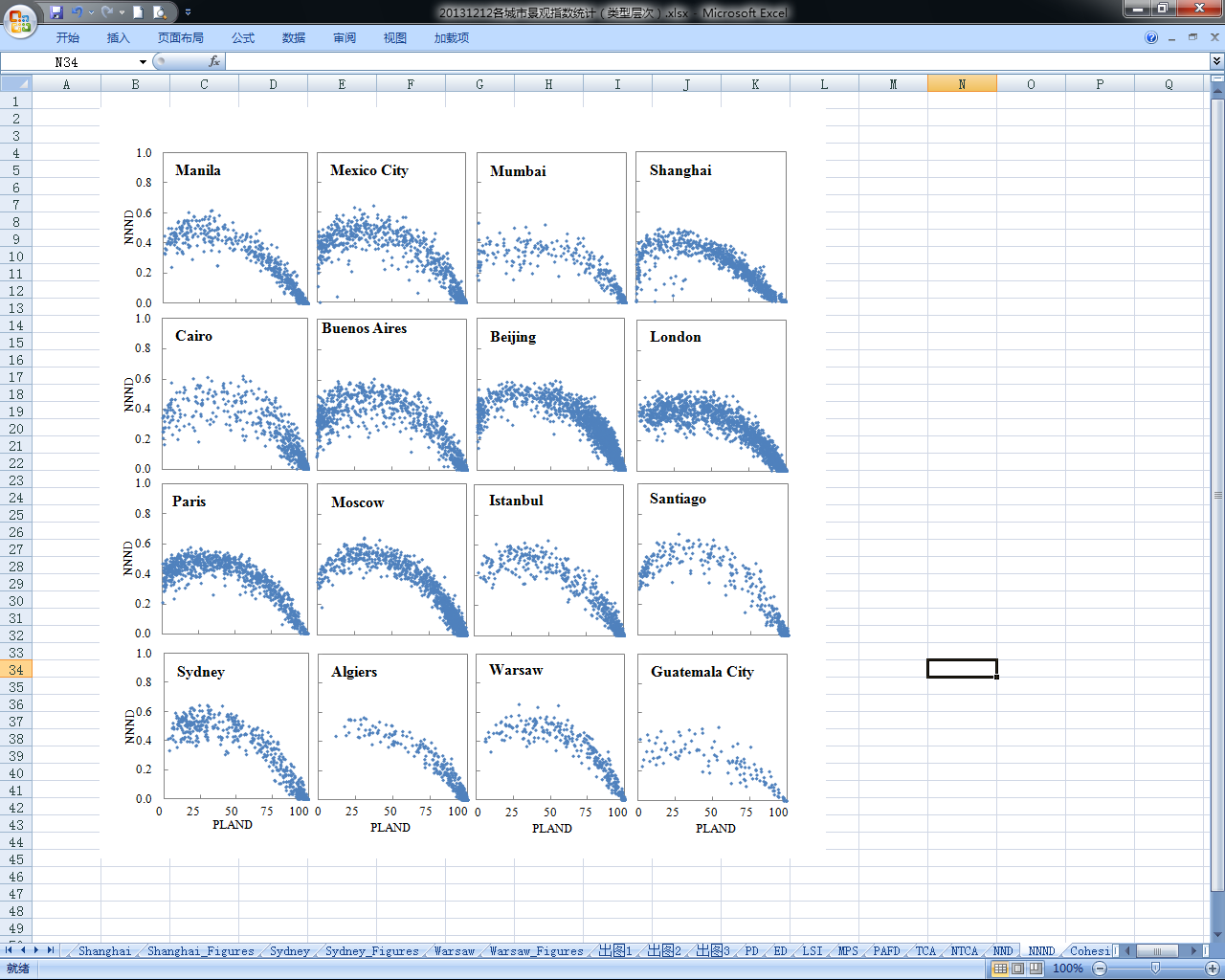


Figure T. The relationship between percentage of habitat (PLAND) and normalized nearest neighbor distance (NNND) of habitat based on space-for-time analysis in 2000 at the extent of 64 by 64 pixels.


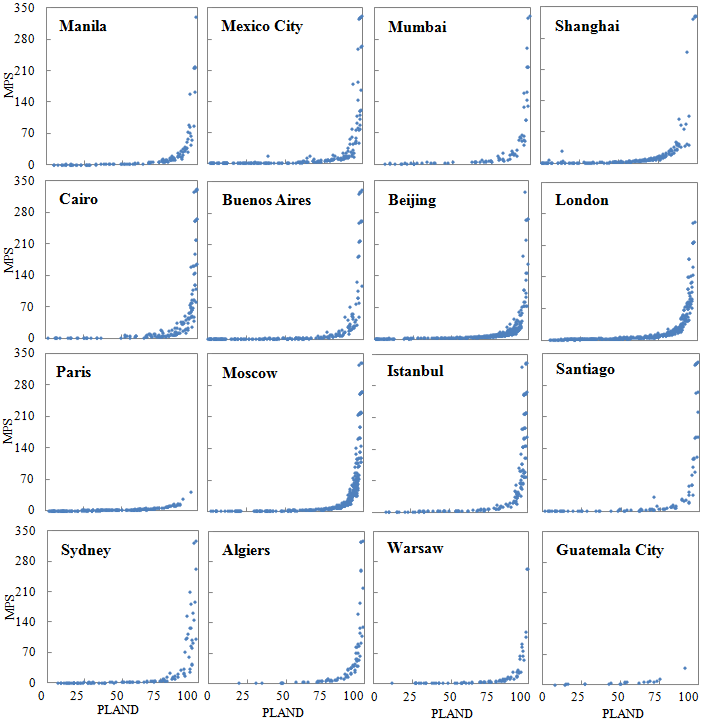


**(a)**

**(b)**


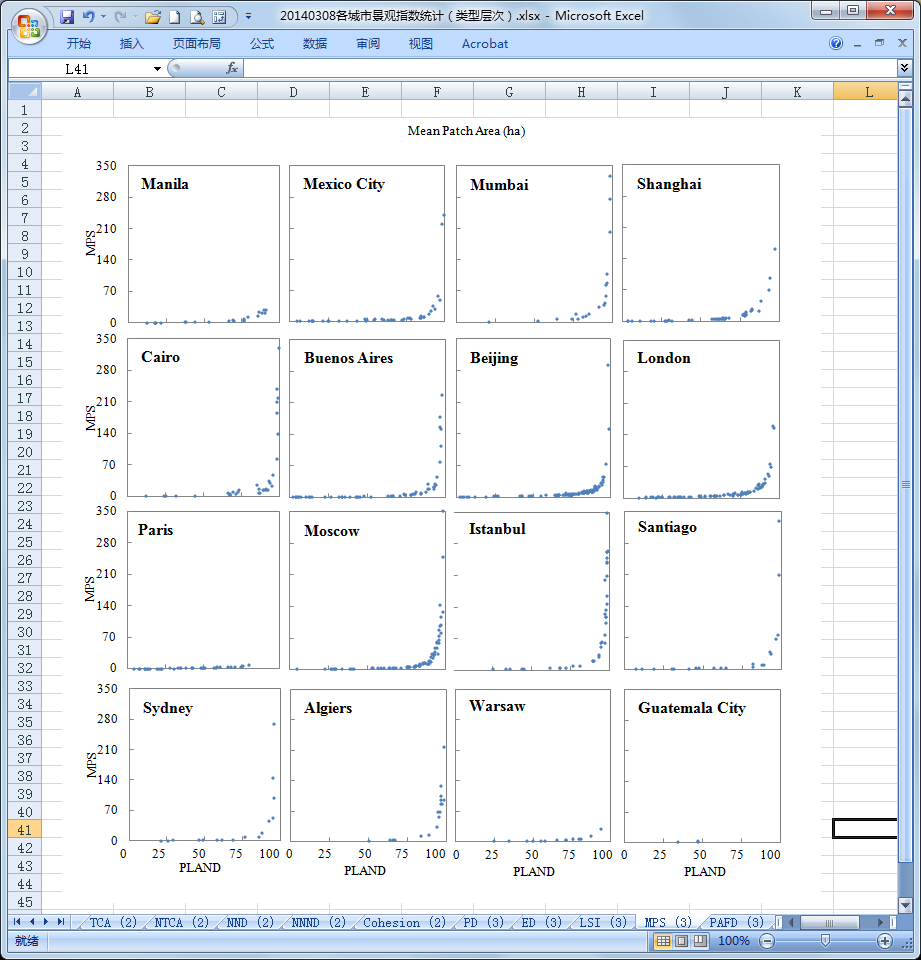


Figure U. The relationship between percentage of habitat (PLAND) and mean patch size (MPS; in ha) of habitat based on space-for-time analysis in 2000 at the extents of 128 by 128 pixels (a) and 256 by 256 pixels (b).


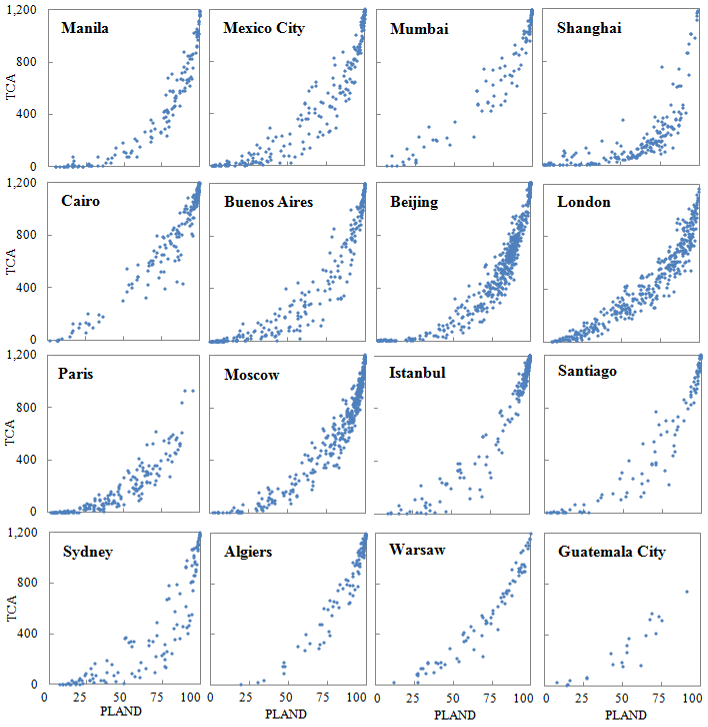


**(a)**

**(b)**


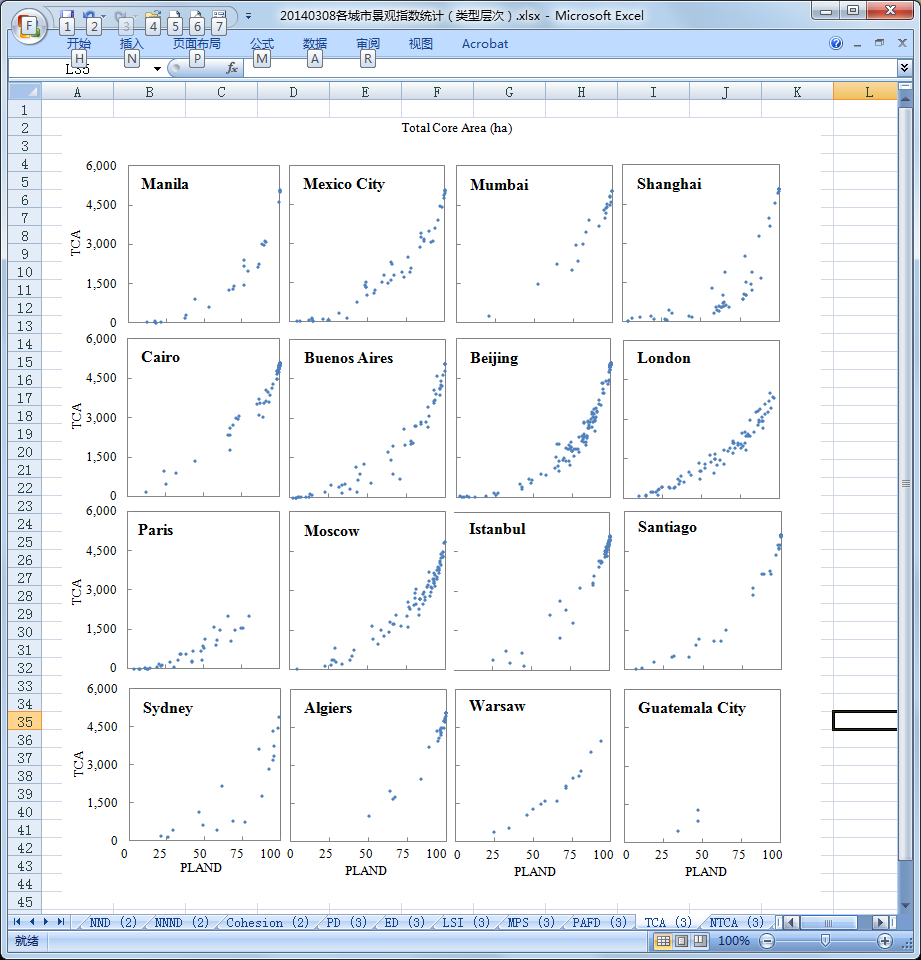


Figure V. The relationship between percentage of habitat (PLAND) and total core area (TCA; in ha) of habitat based on space-for-time analysis in 2000 at the extents of 128 by 128 pixels (a) and 256 by 256 pixels (b).


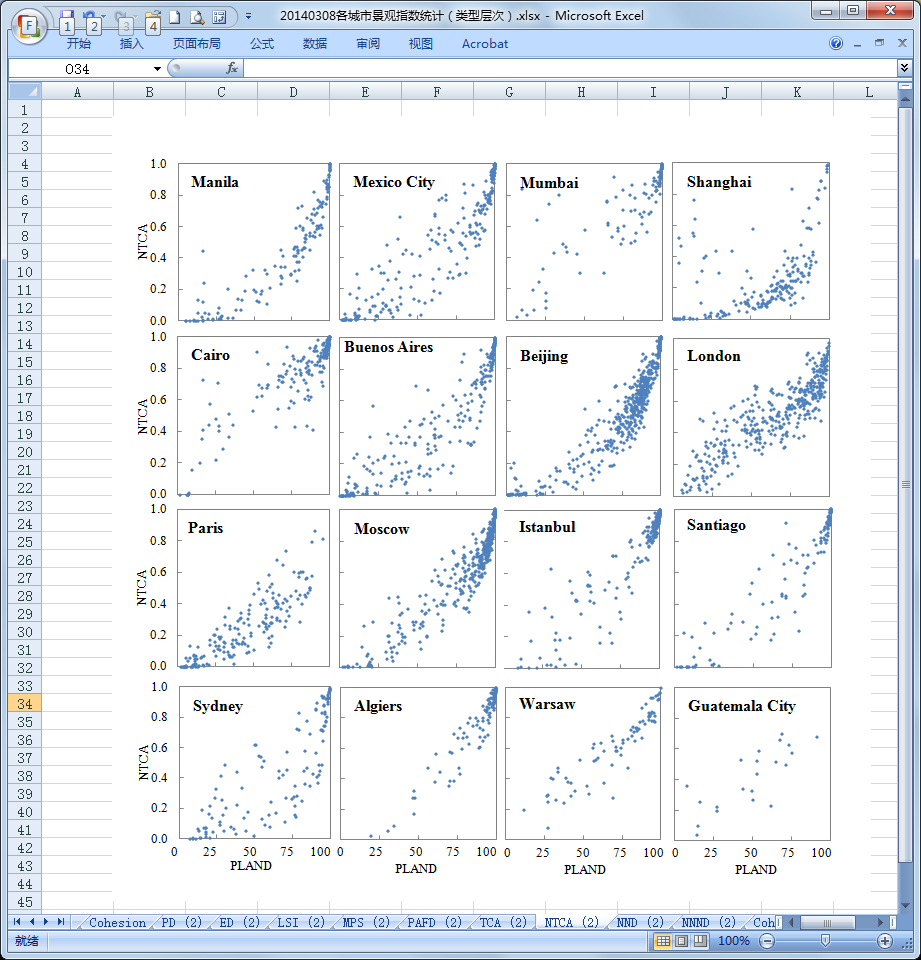


**(a)**

**(b)**


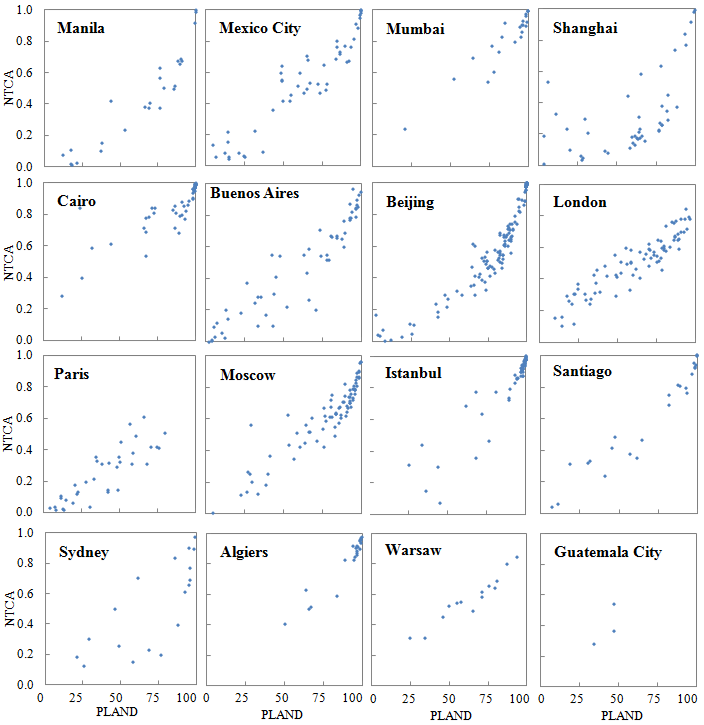


Figure W. The relationship between percentage of habitat (PLAND) and normalized total core area (NTCA) of habitat based on space-for-time analysis in 2000 at the extents of 128 by 128 pixels (a) and 256 by 256 pixels (b).


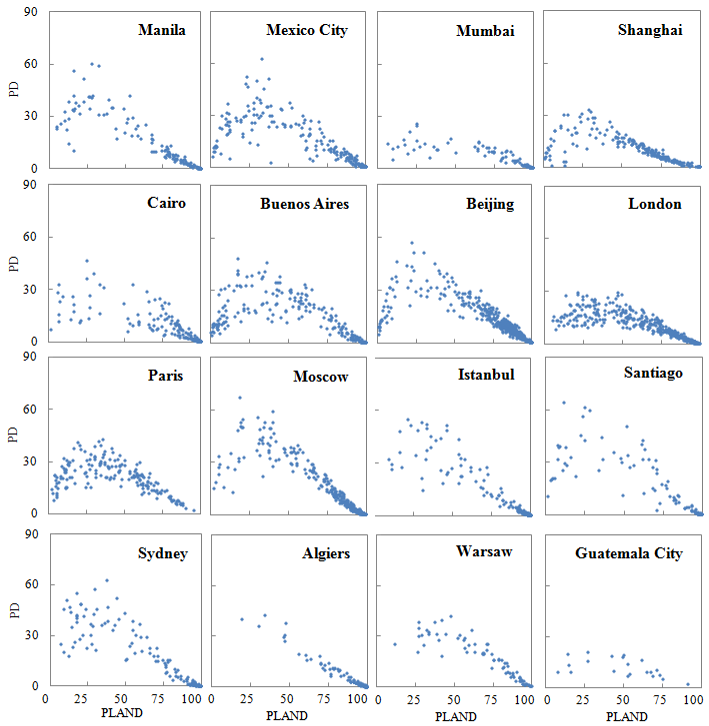


**(a)**

**(b)**


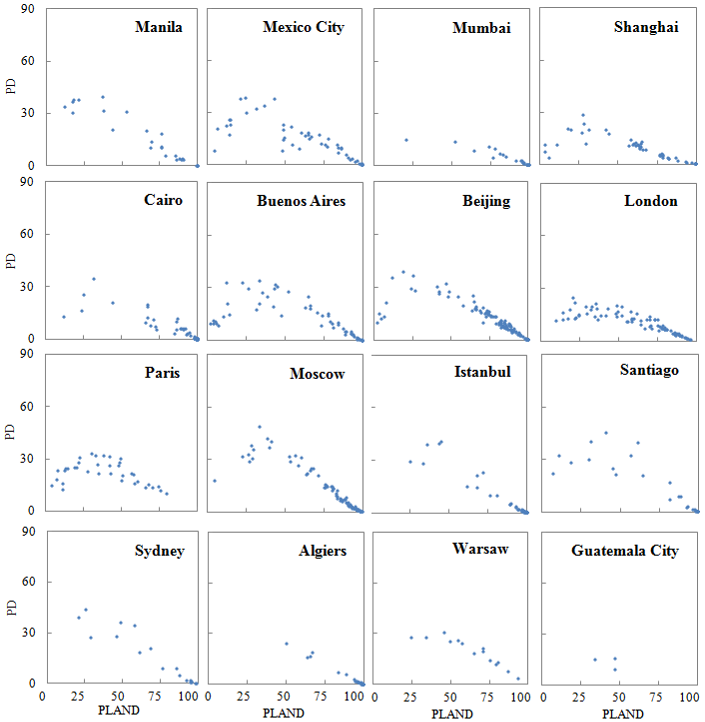


Figure X. The relationship between percentage of habitat (PLAND) and patch density (PD; the number of patches per km^2^) of habitat based on space-for-time analysis in 2000 at the extents of 128 by 128 pixels (a) and 256 by 256 pixels (b).


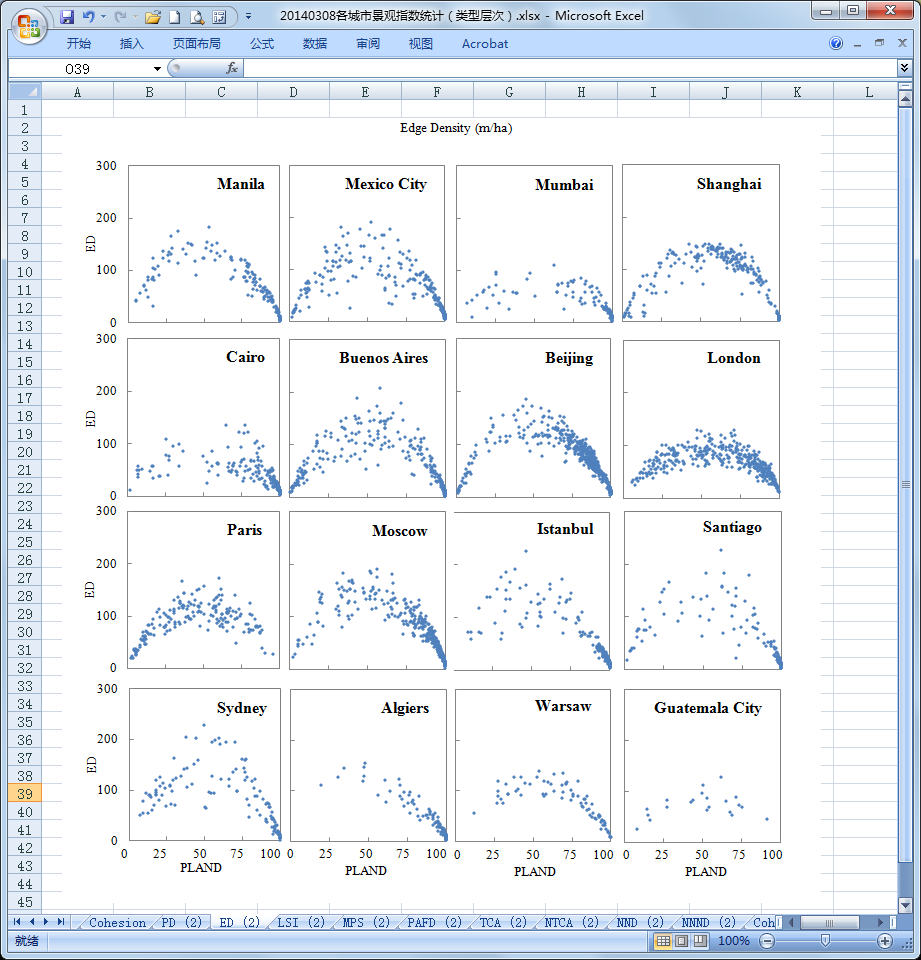


**(a)**

**(b)**


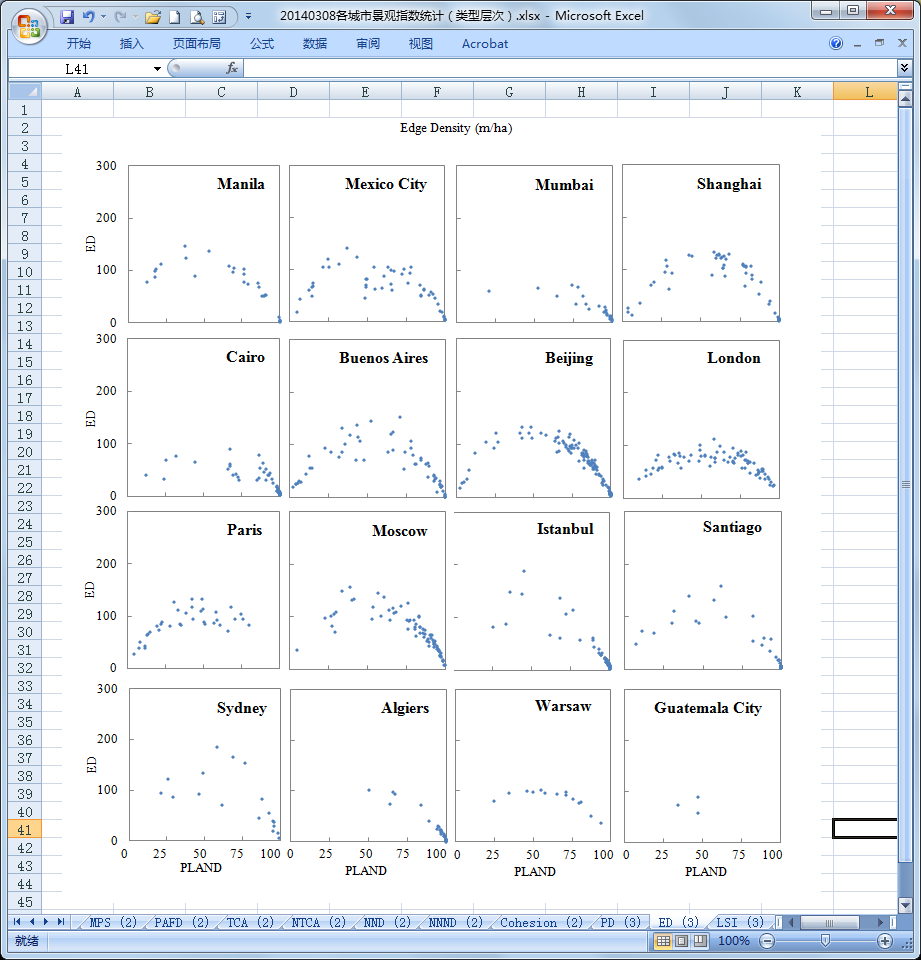


Figure Y. The relationship between percentage of habitat (PLAND) and edge density (ED; the meter per ha) of habitat based on space-for-time analysis in 2000 at the extents of 128 by 128 pixels (a) and 256 by 256 pixels (b).


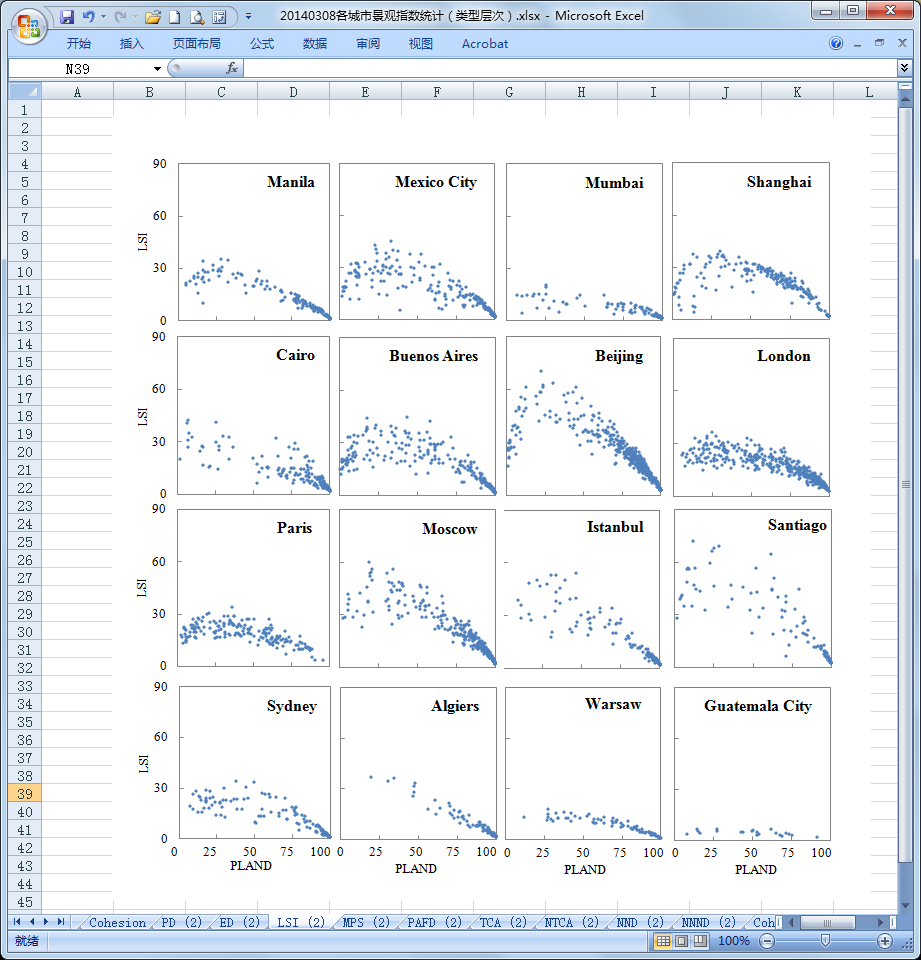


**(a)**

**(b)**


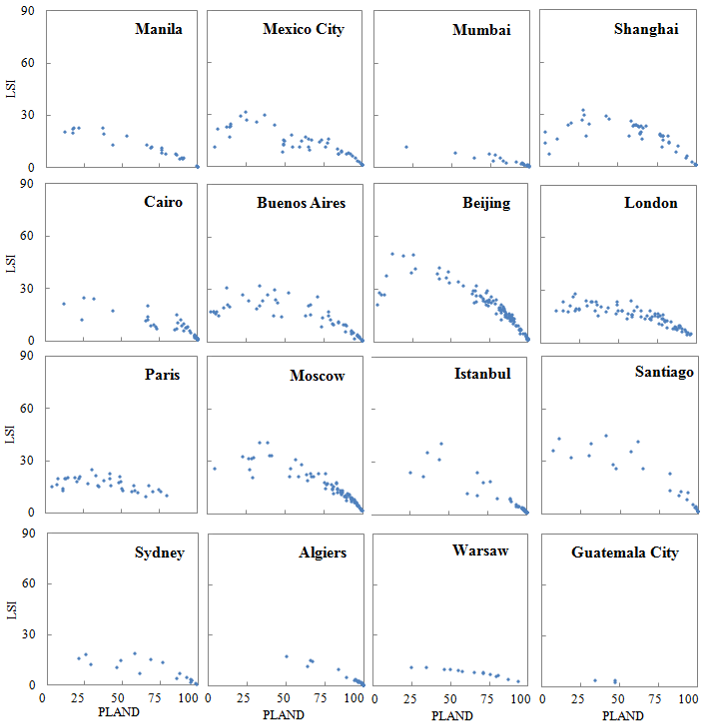


Figure Z. The relationship between percentage of habitat (PLAND) and landscape shape index (LSI) of habitat based on space-for-time analysis in 2000 at the extents of 128 by 128 pixels (a) and 256 by 256 pixels (b).


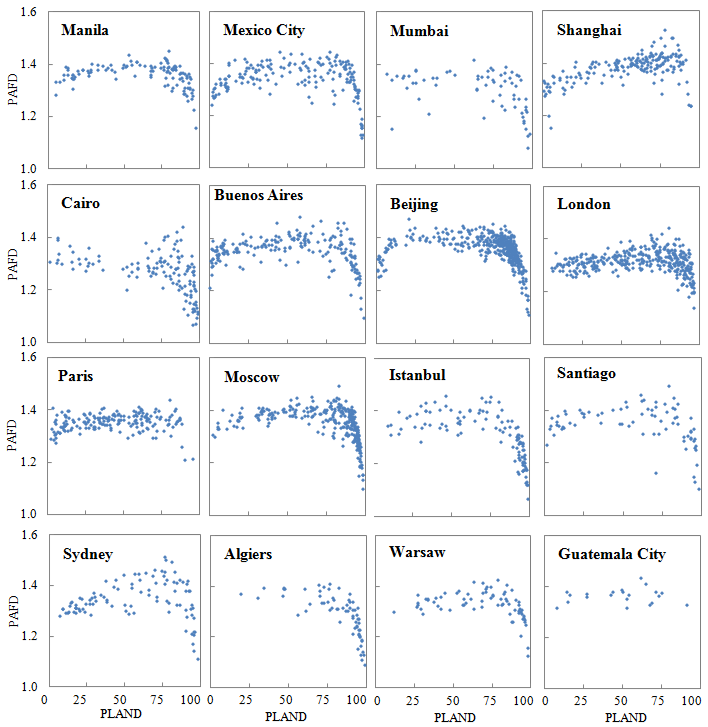


**(a)**

**(b)**


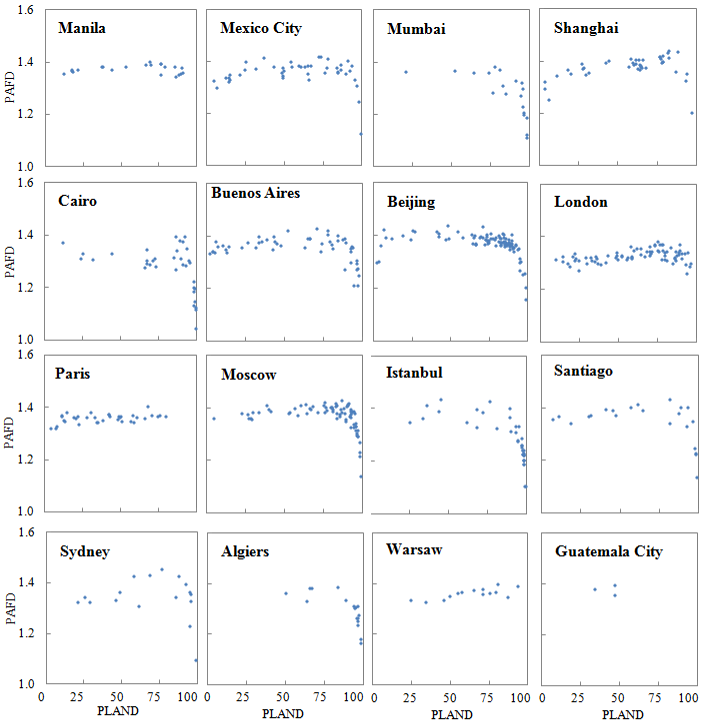


Figure AA. The relationship between percentage of habitat (PLAND) and perimeter-area fractal dimension (PAFD) of habitat based on space-for-time analysis in 2000 at the extents of 128 by 128 pixels (a) and 256 by 256 pixels (b).


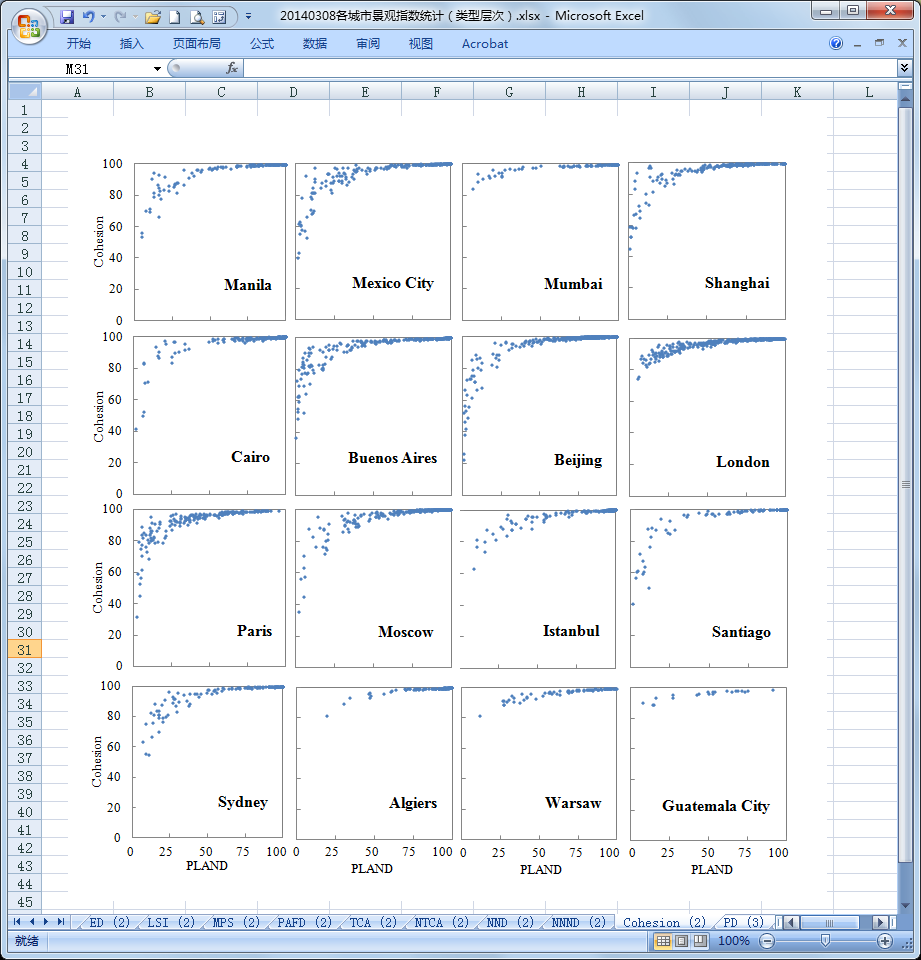


**(a)**

**(b)**


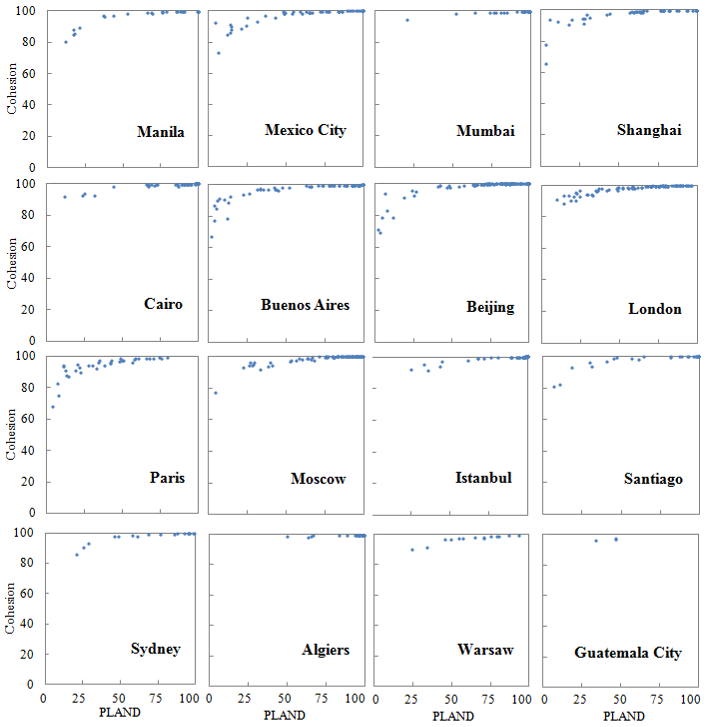


Figure AB. The relationship between percentage of habitat (PLAND) and Cohesion of habitat based on space-for-time analysis in 2000 at the extents of 128 by 128 pixels (a) and 256 by 256 pixels (b).


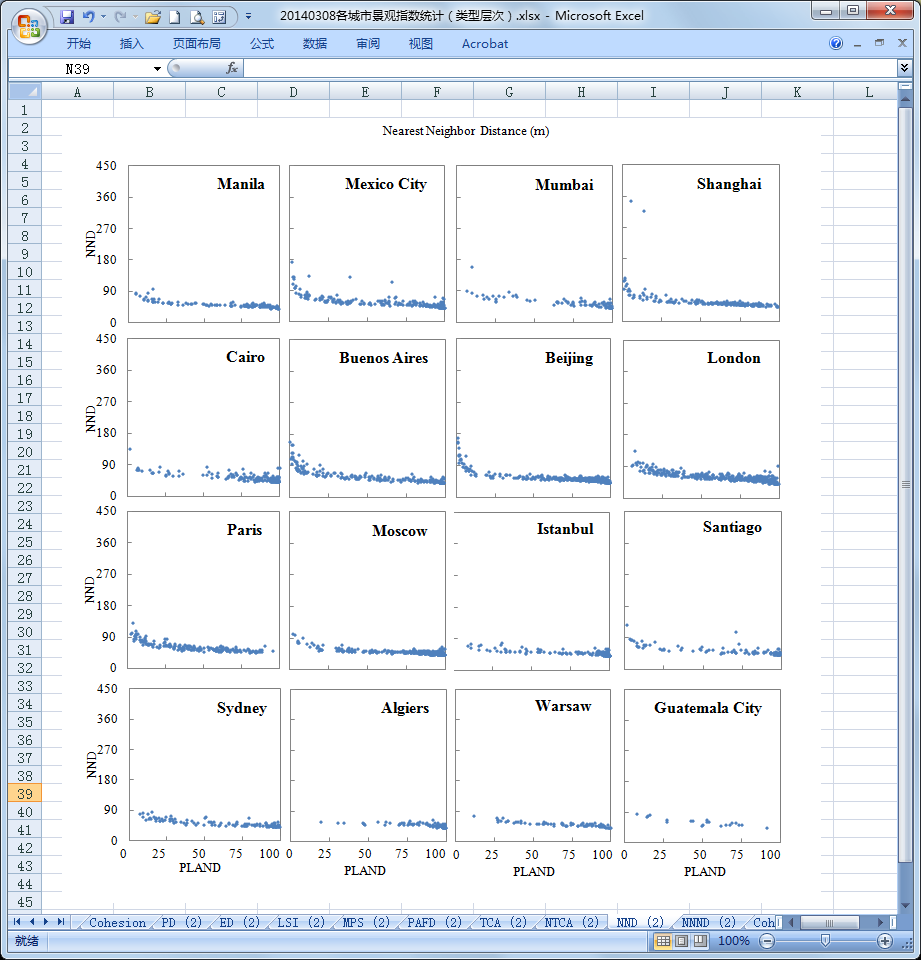


**(a)**

**(b)**


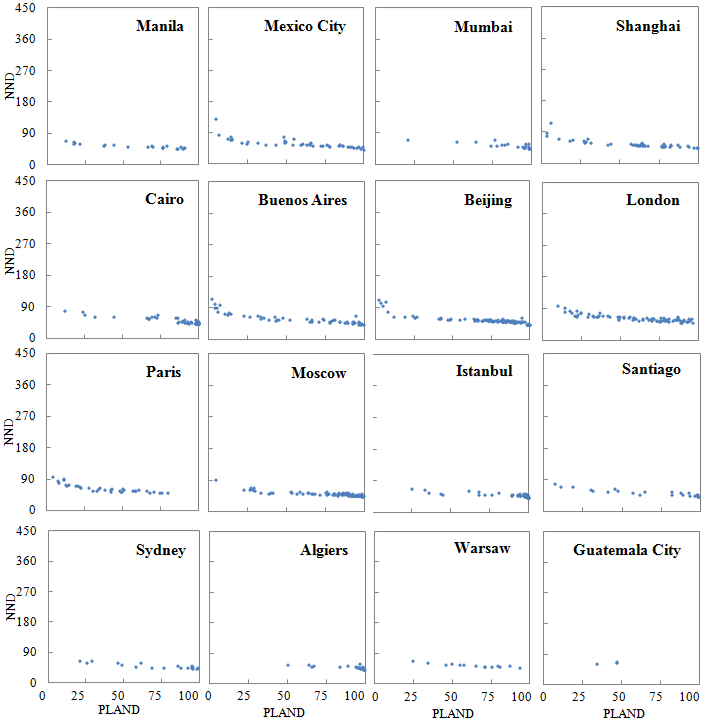


Figure AC. The relationship between percentage of habitat (PLAND) and nearest neighbor distance (NND; in meter) of habitat based on space-for-time analysis in 2000 at the extents of 128 by 128 pixels (a) and 256 by 256 pixels (b).


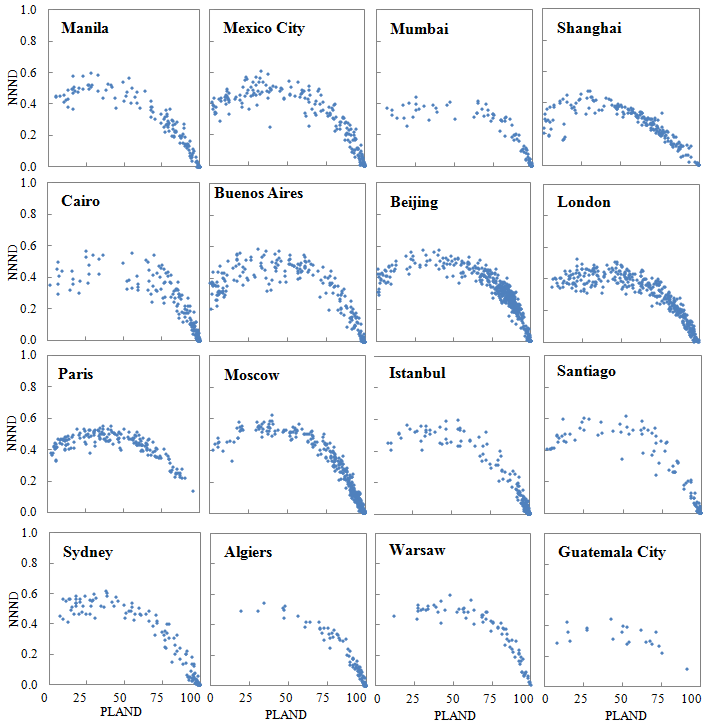


**(a)**

**(b)**


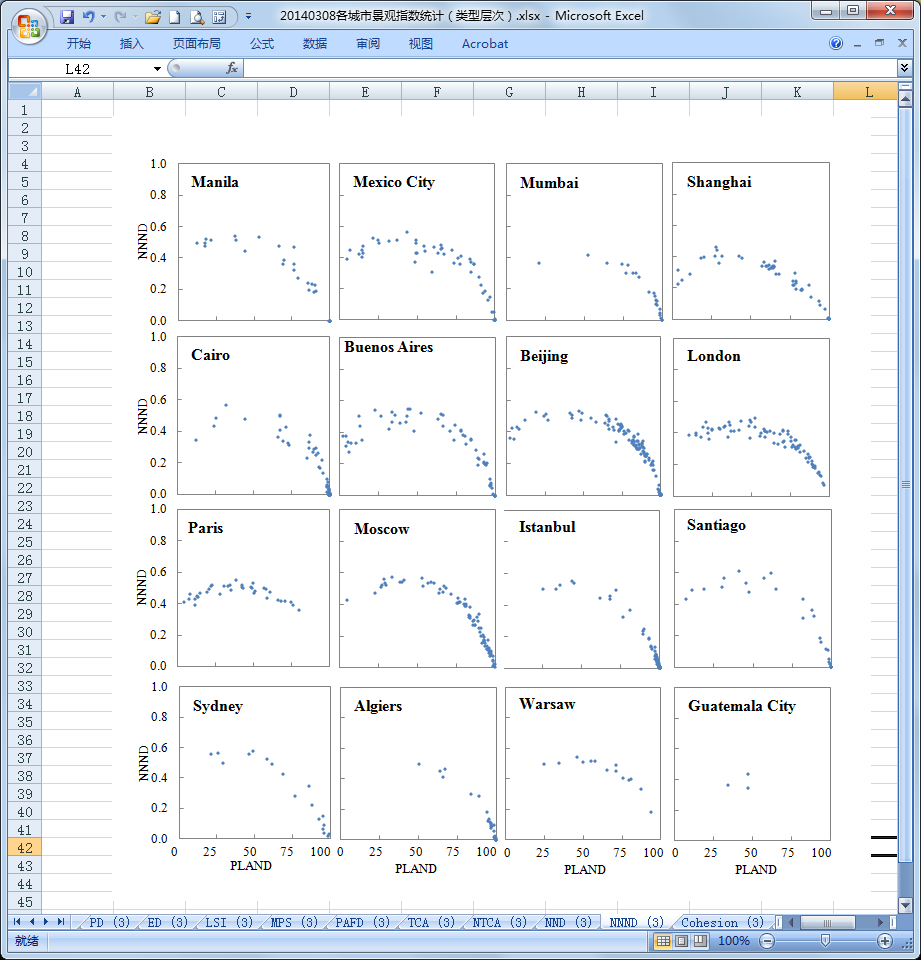


Figure AD. The relationship between percentage of habitat (PLAND) and normalized nearest neighbor distance (NNND) of habitat based on space-for-time analysis in 2000 at the extents of 128 by 128 pixels (a) and 256 by 256 pixels (b).
